# Altered dopamine release and monoamine transporters in Vps35 p.D620N knock-in mice

**DOI:** 10.1038/s41531-018-0063-3

**Published:** 2018-08-21

**Authors:** Stefano Cataldi, Jordan Follett, Jesse D. Fox, Igor Tatarnikov, Chelsie Kadgien, Emil K. Gustavsson, Jaskaran Khinda, Austen J. Milnerwood, Matthew J. Farrer

**Affiliations:** 1 Centre for Applied Neurogenetics, University of British Columbia, Vancouver, Canada; 2 Department of Neurology, St. Olav’s Hospital, Trondheim, Norway

## Abstract

Vacuolar protein sorting 35 (VPS35) is a core component of the retromer trimer required for endosomal membrane-associated protein trafficking. The discovery of a missense mutation, *Vps35* p.D620N implicates retromer dysfunction in the pathogenesis of Parkinson’s disease (PD). We have characterized a knock-in mouse with a *Vps35* p.D620N substitution (hereafter referred to as VKI) at 3 months of age. Standardized behavioral testing did not observe overt movement disorder. Tyrosine hydroxylase (TH)-positive nigral neuron counts and terminal expression in striata were comparable across genotypes. Fast scan cyclic voltammetry revealed increased dopamine release in VKI striatal slices. While extracellular dopamine collected via striatal microdialysis of freely moving animals was comparable across genotypes, the ratio of dopamine metabolites to dopamine suggests increased dopamine turnover in VKI homozygous mice. Western blot of striatal proteins revealed a genotype-dependent decrease in dopamine transporter (DAT) along with an increase in vesicular monoamine transporter 2 (VMAT2), albeit independent of changes in other synaptic markers. The reduction in DAT was further supported by immunohistochemical analysis. The data show that the dopaminergic system of VKI mice is profoundly altered relative to wild-type littermates. We conclude early synaptic dysfunction contributes to age-related pathophysiology in the nigrostriatal system that may lead to parkinsonism in man.

## Neurogenetics: Impaired recycling impacts neurotransmission

A mutation linked to late-onset Parkinson’s disease (PD) disrupts dopaminergic neurotransmission in mice. A study led by Matthew J. Farrer at the Centre for Applied Neurogenetics, University of British Columbia, Canada, examined the effects of a missense mutation in the gene encoding a core subunit of a complex required for recycling membrane-associated proteins, Vacuolar Protein Sorting 35 (VPS35; *Vps35*), in young adult mice. Although no significant effects on motor function or neurodegeneration were found, an increase in dopamine release and turnover in the dorsolateral striatum were observed in the mutant mice. Deficits in the transport and recycling of transporters required for dopamine re-uptake and storage may underlie these effects. Further analyses of *Vps35* mutant mice could improve our understanding of the synaptic dysfunction that precedes neurodegeneration in PD.

## Introduction

Parkinson’s disease (PD) is an age-related neurodegenerative disease characterized by the loss of midbrain dopaminergic neurons and Lewy pathology.^1^ However, earlier loss of dopaminergic markers in the striatum suggests synaptic alterations and axonal denervation may precede nigral neuronal degeneration.^2^ Movement disorder occurs insidiously, characterized by tremor, rigidity, bradykinesia, and postural instability. Non-motor dysfunction including autonomic, cognitive, psychiatric, sensory, and sleep disorders are also associated with motor symptoms and typically antedate them.^3^ While dopamine-replacement therapies alleviate motor symptoms, they fail to halt disease progression and often lead to dyskinesia. Thus, new therapeutic targets and strategies to combat neuronal cell loss are necessary.

Dominantly-inherited, late-onset parkinsonism has recently been linked to an aspartate to asparagine substitution in vacuolar protein sorting 35 (VPS35 p.D620N).^4,5^ Affected subjects in >61 unrelated families have been described with a clinical syndrome indistinguishable from idiopathic PD.^6^ VPS35, VPS26, and VPS29 are structural components of the retromer trimer^7,8^ that complex with a pair of sorting nexins to recycle transmembrane cargo to the cell surface or *trans-*Golgi network.^9^ Within the synapse, the retromer recycles neurotransmitter receptors including β-adrenergic, AMPA-type glutamate, and D1-type dopamine receptors.^10–15^

A line of *Vps35* p.D620N knock-in mice was recently reported without a behavioral phenotype, although striatal microdialysis revealed a reduction in evoked striatal dopamine in homozygous animals.^16^ Here we report an independent *Vps35* p.D620N knock-in mouse model (VKI) developed to preserve the physiological 1:1:1 stoichiometry of the retromer complex. Given the selective vulnerability and loss of dopamine neurons in PD, we hypothesized *Vps35* p.D620N may impair retromer-dependent recycling of specific cargo with consequent effects on dopaminergic neurotransmission. Hence, at 3 months of age, our characterization has focused on motor behavior, the integrity of the nigrostriatal system, and dopamine release.

## Results

### *Vps35* p.D620N knock-in mice

**Fig. 1:**
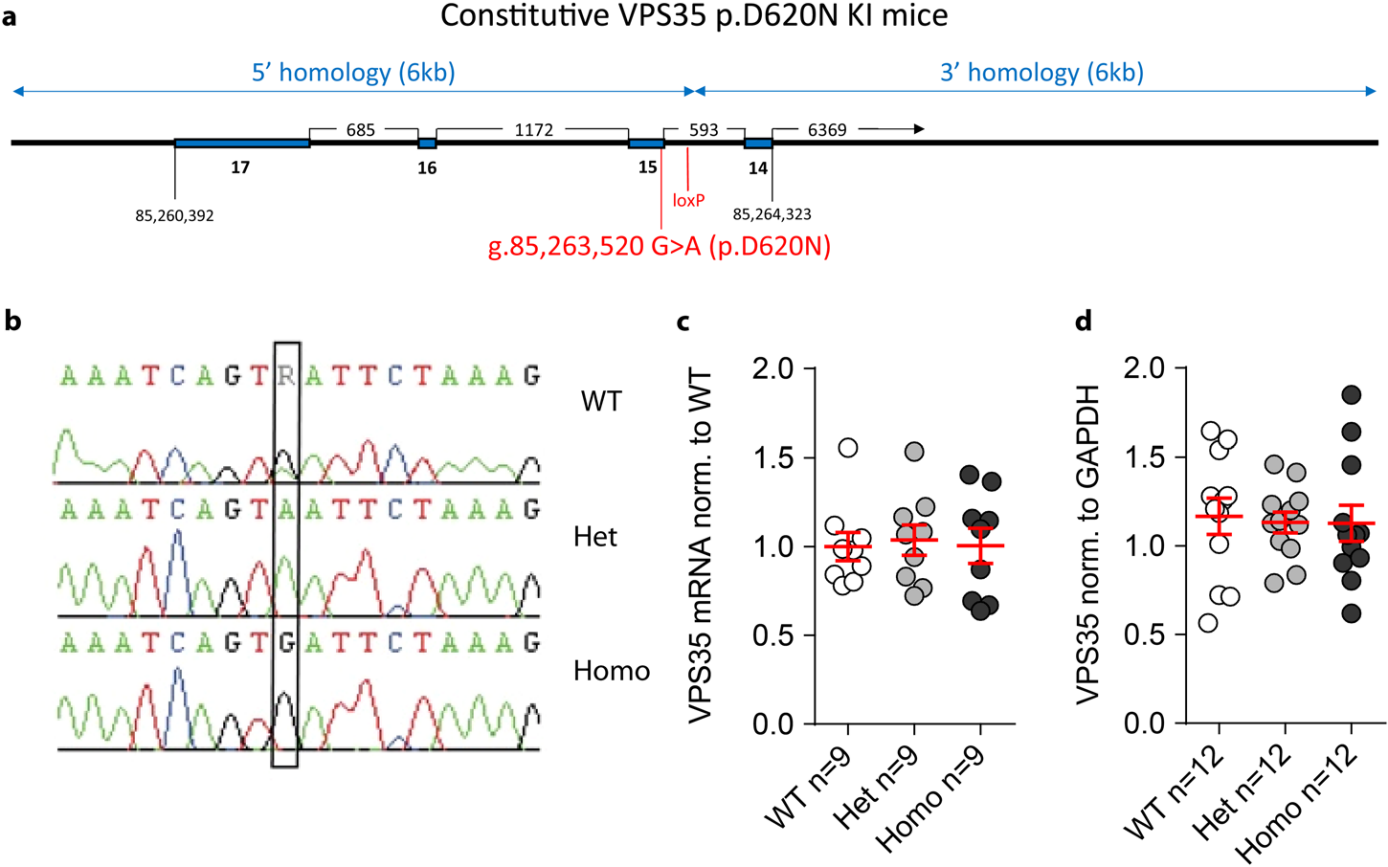
Generation of Vps35 p.D620N knock-in mice (VKI). **a** Schematic of the targeting design showing the murine *Vps35* genomic sequence (Ensembl reference ENSMUSG00000031696), 5′ and 3′ homology arms (arrowed), exons 14–17 (blue), engineered loxP site in intron 14, the g.85,263,520G>A (p.D620N) mutation in exon 15 (encoding p.D620N) and endogenous stop codon in exon 17 (TAA). FlpE-recombinase deletion between FRT sites removed the *PKG*-neo-pA cassette to yield the cVKI allele (not shown). Subsequent Cre-recombinase deletion between loxP sites created the VKI allele. **b** cDNA sequencing in wild-type mice (top), heterozygous VKI (middle), and homozygous VKI (bottom), between g.8:85263511–85263528 (GRCm38) highlights the g.85,263,520G>A nucleotide mutation in exon 15 that encodes the p.D620N amino acid substitution. **c** Relative *Vps35* mRNA (fold change) expression analysis in cerebellar tissue using quantitative RT-PCR. Data normalized to WT (1-way ANOVA *F*_2,24_ = 0.05, *p* *=* 0.95). **d** Total Vps35 protein levels were quantified by western blotting, showing no difference in Het and Homo mice compared to their WT littermates (1-way ANOVA *F*_2,33_ = 0.05, *p* = 0.94)

To examine the effects of the *Vps35* p.D620N mutation at physiological levels, and avoid the confounds of random insertion and overexpression, we engineered C57Bl/6J mice to express a *Vps35* exon 15 g.85,263,520G>A (p.D620N) nucleotide mutation (Fig. 1a). Successful manipulation was verified by genomic and cDNA sequencing (Fig. 1b). Wild-type (WT) and mutant allele-specific *Vps35* mRNA expression was equimolar and unaltered by gene targeting in VKI animals (Fig. 1c, 1-way ANOVA *p* = 0.95). Similarly, total *Vps35* protein levels were unchanged (Fig. 1d, 1-way ANOVA *p* = 0.94). VKI mice are viable, show no evidence of overt distress, breed well and produce heterozygous (Het) and homozygous (Homo) pups at expected Mendelian ratios.

### VKI mice have normal body weight, locomotor function, and show no nigral neuronal loss

**Fig. 2:**
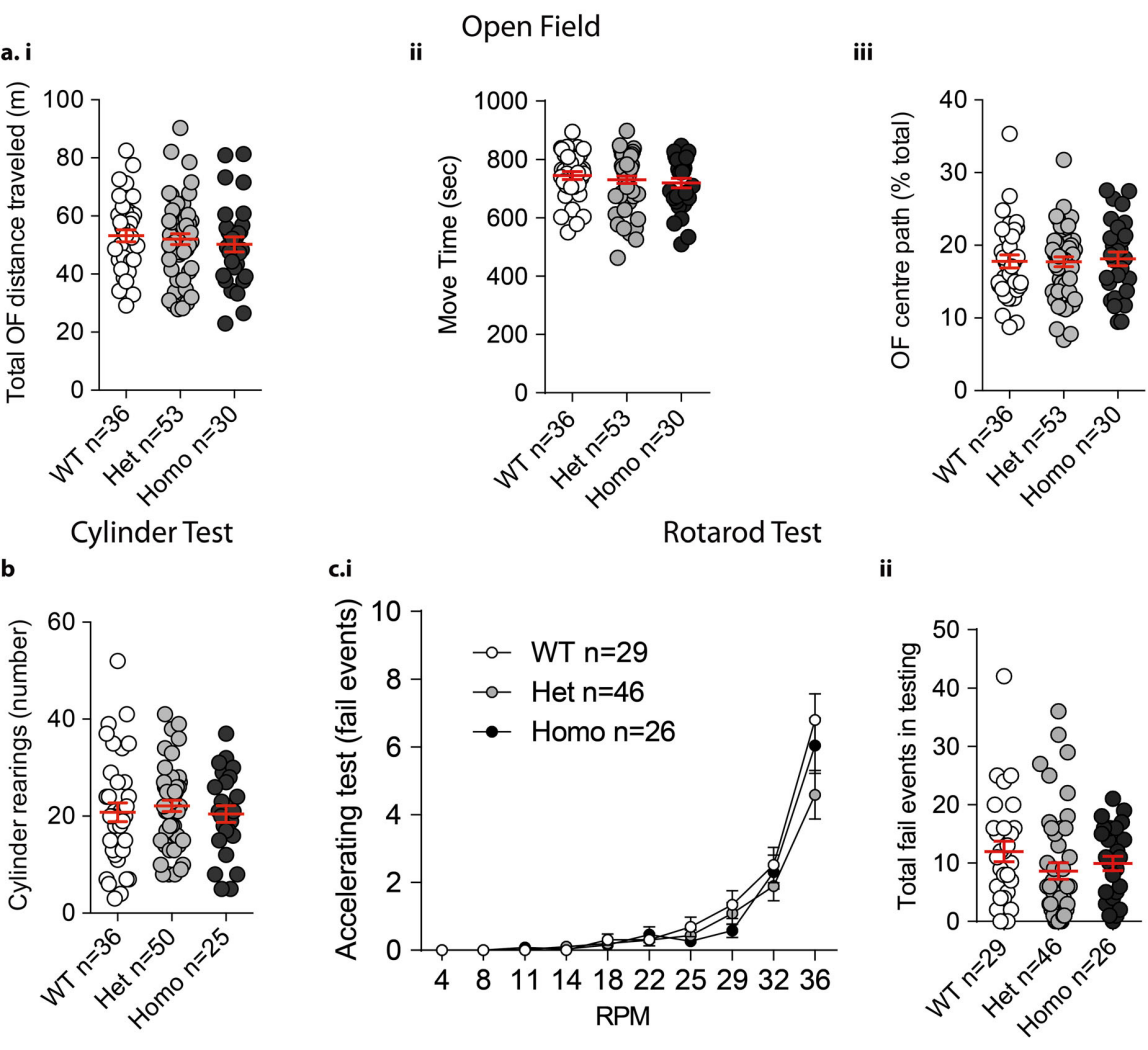
Behavioral evaluation of VKI mice. **a** Mice were placed in an open field arena and video recorded as they explored for 15 min. Total distance traveled and moving time was not different across genotypes (i and ii, 1-way ANOVA *F*_2,116_ = 0.39, *p* *=* 0.67 and *F*_2,116_ = 0.71, *p* = 0.49, respectively). Percentage of central path exploration was also similar across genotypes (iii, 1-way ANOVA *F*_2,116_ = 0.06, *p* = 0.94). **b** Mice were video recorded for 5 min after being placed in a 1 L-glass beaker. The number of rearings was scored off-line by an experimenter blinded to genotype. Mice show no significant difference in novelty-induced vertical exploration (rearing; 1-way ANOVA *F*_2,109_ = 0.33 *p* = 0.71). **c** Mice were placed on an accelerating rotarod (from 4 to 36 RPM) for 5 min. A fall or hold on the rotarod for a revolution was considered a fail event. VKI mice show no differences in their performance when compared with their WT littermates (i and ii, fail per acceleration: 2-way ANOVA interaction *F*_18,882_ = 1.73, *p* = 0.03, RPM *F*_9,882_ = 104.9, *p* < 0.0001, genotype *F*_2,98_ = 1.28, *p* = 0.28, subjects *F*_98,882_ = 2.58, *p* < 0.0001, and total fails during testing: 1-way ANOVA *F*_2,100_ = 1.45, *p* = 0.24, respectively)

At 3 months there were no differences in appearance or weight of VKI mice when compared to WT littermates (Supp. Fig. 1, 1-way ANOVA *p* = 0.76). Basic motor function was normal in VKI as compared to WT littermates. There were no changes in open field exploration, as shown by similar total distance traveled, moving time (Fig. 2a.i,ii, 1-way ANOVA *p* = 0.67 and *p* = 0.49, respectively) and center path ratio (Fig. 2a.iii, 1-way ANOVA *p* = 0.93). Similarly, there were no differences in cylinder test rearing activity (Fig. 2b, 1-way ANOVA *p* = 0.71) or performance on an accelerating rotarod (Fig. 2c.i,ii, 1-way ANOVA *p* = 0.88 and *p* = 0.14, respectively).

**Fig. 3:**
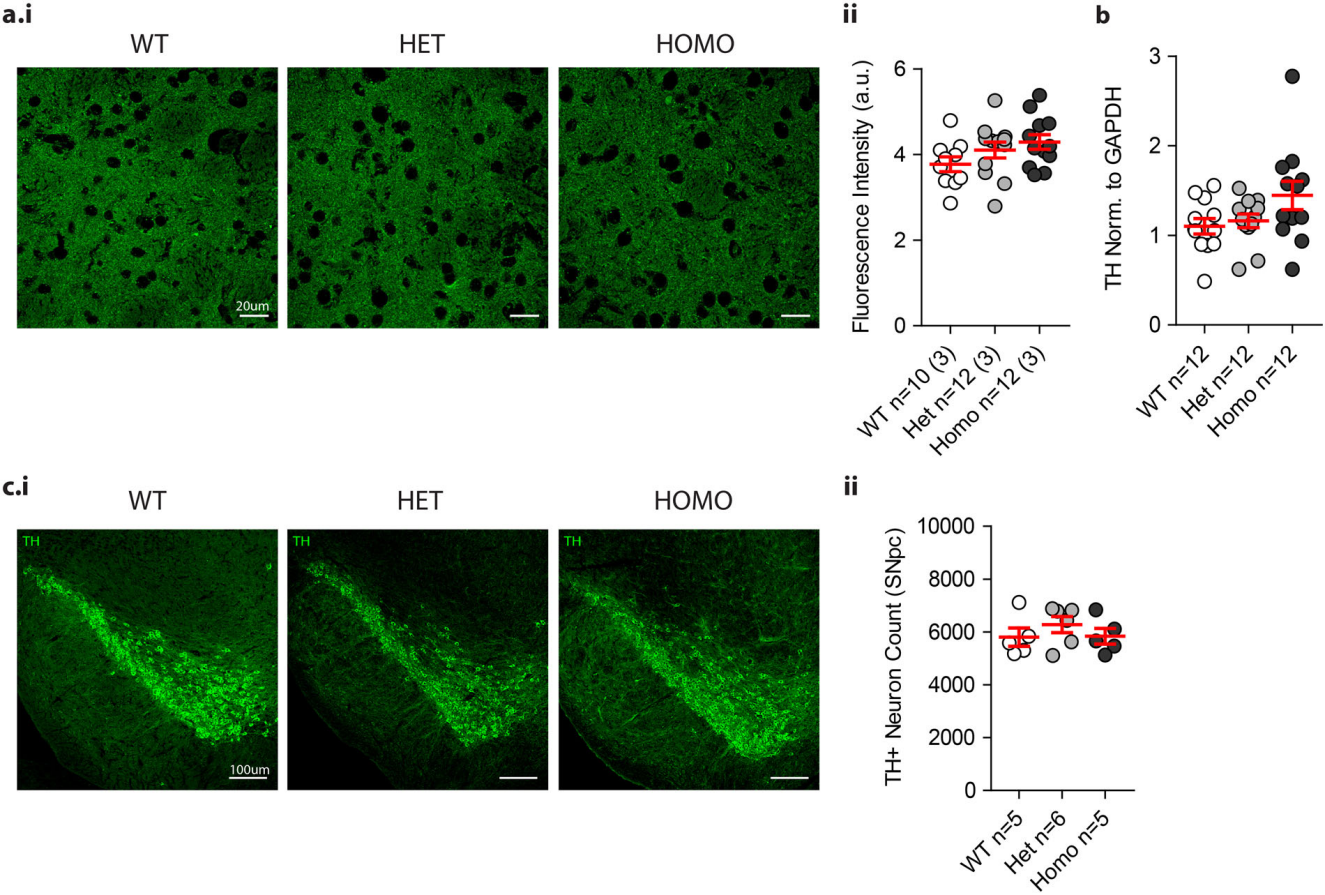
Evaluation of nigrostriatal TH-positive neurons in VKI mice. Representative images of TH (green) staining in striatal (**a**.i.) and nigral (**c**.i) sections of PFA perfused brain from VKI mice. No difference was detected in total levels of TH in the striatum (**a**.ii, 1-way ANOVA *F*_2,6_ = 0.28, *p* = 0.75) across genotypes, nor in the total count of TH-positive neurons in the SNpc (**c**.ii, 1-way ANOVA *F*_2,13_ = 0.74, *p* = 0.49) of the same mice. Similarly there were no differences in total level of TH in the striatum quantified with Western blotting (**b**, 1-way ANOVA *F*_2,33_ = 2.86, *p* = 0.08) shown cropped (for full-length blot see Supp. Fig. 2)

We examined the integrity of the nigrostriatal system [*corpus striatum* (striatum) and *substantia nigra pars compacta* (SNpc)] by immunohistochemical staining of the dopamine synthesis enzyme tyrosine hydroxylase (TH). No significant differences were observed between VKI and WT littermates in the intensity of TH-signal produced by nigral terminals in the dorsolateral striatum (Fig. 3a.ii, 1-way ANOVA *p* = 0.75), or in the number of TH-positive nigral neurons assessed by stereological counts (Fig. 3c.ii, 1-way ANOVA *p* = 0.49). Consistent findings were observed by western blotting of striatal tissue probed for TH (Fig. 3b, 1-way ANOVA *p* = 0.08). Together, the data indicate that physiological expression of *Vps35* p.D620N does not overtly impair motor function or induce dopaminergic neurodegeneration in young adult mice.

### Striatal dopamine release

**Fig. 4:**
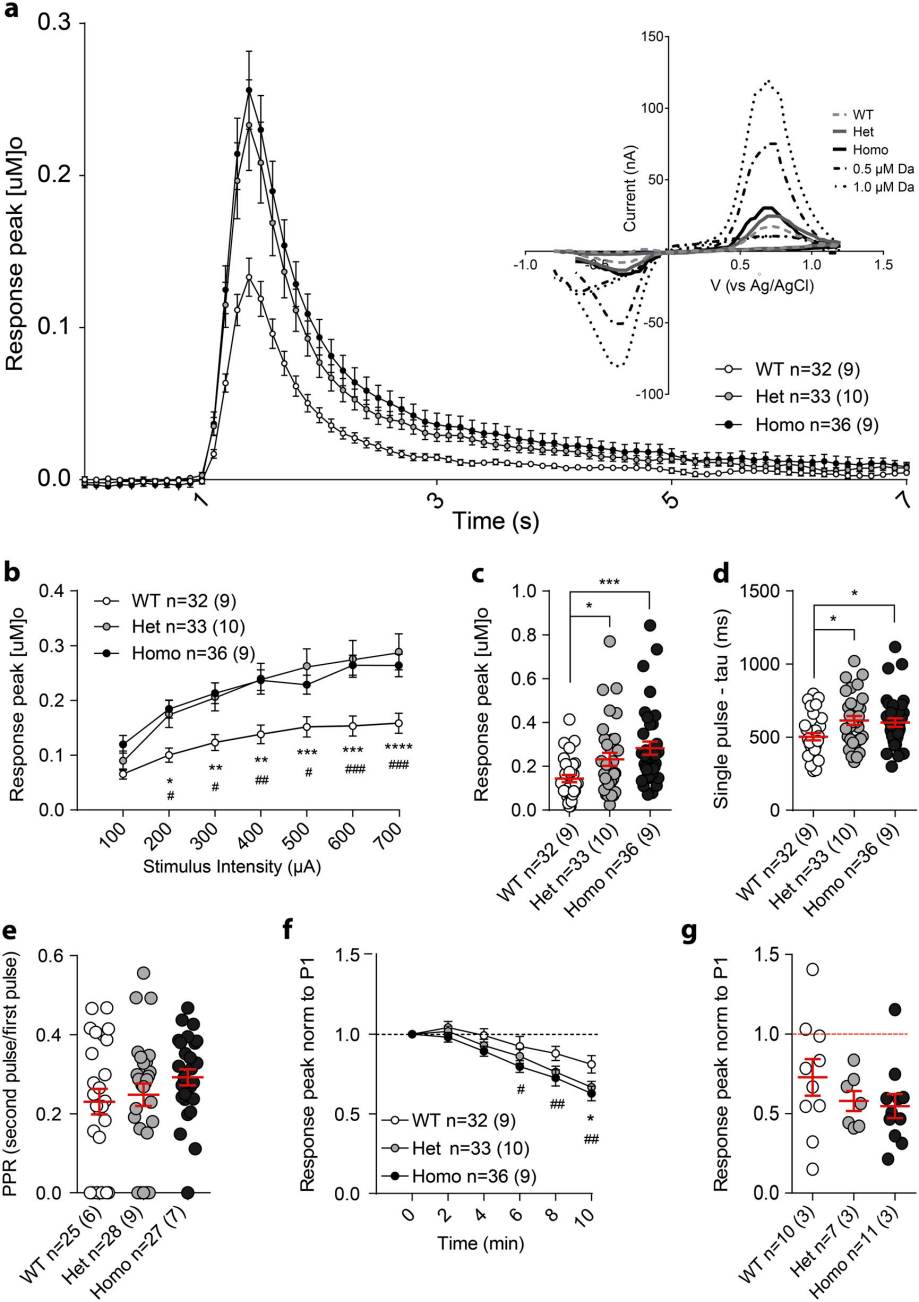
Fast scan cyclic voltammetry of evoked extracellular dopamine release and reuptake. **a** Example of average peak for each genotype, indicating greater response and slower decay in slices from VKI animals. On the top right corner, examples of the voltammograms for dopamine detection showing oxidation and reduction of dopamine, one for each genotype and calibrations (0.5 and 1 µM) used at the end of the recording each day. **b** A series of increasing intensity single pulse stimuli were delivered every 2 min to obtain an input/output curve. VKI slices showed an increase in evoked dopamine as compared to slices from their WT littermates (2-way ANOVA interaction *F*_12,588_ = 3.871, *p* < 0.0001, input *F*_6,588_ = 87.54, *p* < 0.0001, genotype *F*_2,98_ = 7.097, *p* < 0.05, subject *F*_98,588_ = 29.35, *p* < 0.0001; Bonferroni post-test WT vs Het ^*^p < 0.05, ^**^p < 0.01^***^, *p* < 0.001^****^, *p* < 0.0001, WT vs Homo; ^#^*p* < 0.05, ^##^*p* < 0.01, ^###^*p* < 0.001). **c** At stimulation intensities leading to 50–70% maximum dopamine release, based on the input/output, a series of 3 pulses were given with an interval of 2 min between each. With this paradigm the single pulse average was also greater in slices from Het and Homo mice (1-way ANOVA *F*_2,59_ = 6.937, *p* < 0.05, Bonferroni post-test WT vs Het *t*_(98)_ = 2.284, *p* < 0.05, WT vs Homo *t*_(98)_ = 3.70, *p* < 0.001). **d** Decay times were slower in Het and Homo VKI slices (1-way ANOVA *F*_2,98_ = 4.373, *p* < 0.02, Bonferroni post-test *t*_(98)_ *=* 2.706 *p* < 0.05, and *t*_(98)_ = 2.424, *p* < 0.05 for Het and Homo VKI slices, respectively). **e** VKI slices were comparable to WT in paired-pulse recordings applied 4 s apart (1-way ANOVA *F*_2,77_ = 1.36, *p* = 0.26). **f** Application of the D2 agonist quinpirole (50 nM) in WT slices resulted in a 20–50% reduction in dopamine release within ~5 min. VKI slices showed more rapid D2 inhibition compared to slices from WT littermates (2-way RM-ANOVA, interaction *F*_10,470_ = 1.47, *p* = 1.42, time *F*_5,470_ = 49.24, *p* < 0.0001, genotype *F*_2,94_ = 3.769, *p* < 0.05, subjects *F*_94,470_ = 4.934; Bonferroni post-test WT vs Het ^*^p < 0.05, WT vs Homo; ^#^*p* < 0.05^##^, *p* < 0.01). **g** In a separate cohort, quinpirole (50 nM) treatment for 30 min gave a comparable 40–50% reduction for all genotypes (1-way ANOVA *F*_2,25_ = 1.166, *p* = 0.33)

In acute brain slices, nigrostriatal dopamine release was directly assessed by fast scan cyclic voltammetry (FSCV; Fig. 4 and Supp. Fig. 3) in the dorsolateral striatum. Single stimuli evoked dopamine release, and increased proportionally to stimulus intensity (100–700 µA). In order to examine discrete release, to potentially uncover subtle differences between genotypes, we used a short stimulus duration (150 µs) at sites quite distal (200 µm) to the recording site, similar to those used for electrophysiological recording of striatal field responses (as in ref.^17^). This renders the magnitude of our evoked transients smaller than many in the literature. Nevertheless, we demonstrate that increased stimulus duration and more proximal stimulation produces much larger responses in the same slices (Supp. Fig. 3e.i,ii). The genotype of the animal had a significant effect on input–output relationships with elevated dopamine release in VKI slices (2-way RM-ANOVA genotype *p*<0.01; with Bonferroni post-test as indicated in Fig. 4b). Release evoked by a single stimulation at 50–70% of the maximal response, repeated three times and averaged, was also significantly elevated in VKI slices (Fig. 4c, 1-way ANOVA *p*<0.01, Bonferroni post-test *p*<0.05 and *p*<0.001 from Het and Homo mice, respectively; average traces shown in Fig. 4a). Decay times for these dopamine transients were also significantly slower in VKI slices, suggesting slower re-uptake of extracellular dopamine (Fig. 4d, 1-way ANOVA *p*<0.05, Bonferroni post-test *p*<0.05, *p*<0.05 for Het and Homo, respectively). Dopamine re-release was assayed by paired-pulse experiments; paired stimuli (4 s inter-pulse-interval; IPI) evoked an ~80% depression of the second pulse but there was no difference between genotypes (Fig. 4e, 1-way ANOVA *p* = 0.26). The increase in response amplitude following a stimulation train (1 s at 100 Hz), relative to a single pulse, was equivalent across genotypes (Supp. Fig. 3c, 1-way ANOVA *p*=0.82). Decay times were also increased following a stimulation train (~2-fold), with a significant main genotype effect (Supp. Fig. 3d, 1-way ANOVA *p*<0.05) although groups were not independently different by post hoc test.

Quinpirole, a D2 agonist, was used to investigate D2 auto-receptor activation (50 nM, as previous^17^). Over repeated stimuli applied at 2 min intervals, VKI slices showed a more rapid inhibition of dopamine release when compared to slices from WT littermates (at 10 min 2-way RM-ANOVA, genotype *p* < 0.05; with Bonferroni post-test as indicated in Fig. 4f). However, WT slices reached a similar degree of inhibition as VKI over 30 mins of quinpirole exposure (Fig. 4g, 1-way ANOVA *p* = 0.32).

### Dopamine tissue content in VKI mice

**Fig. 5:**
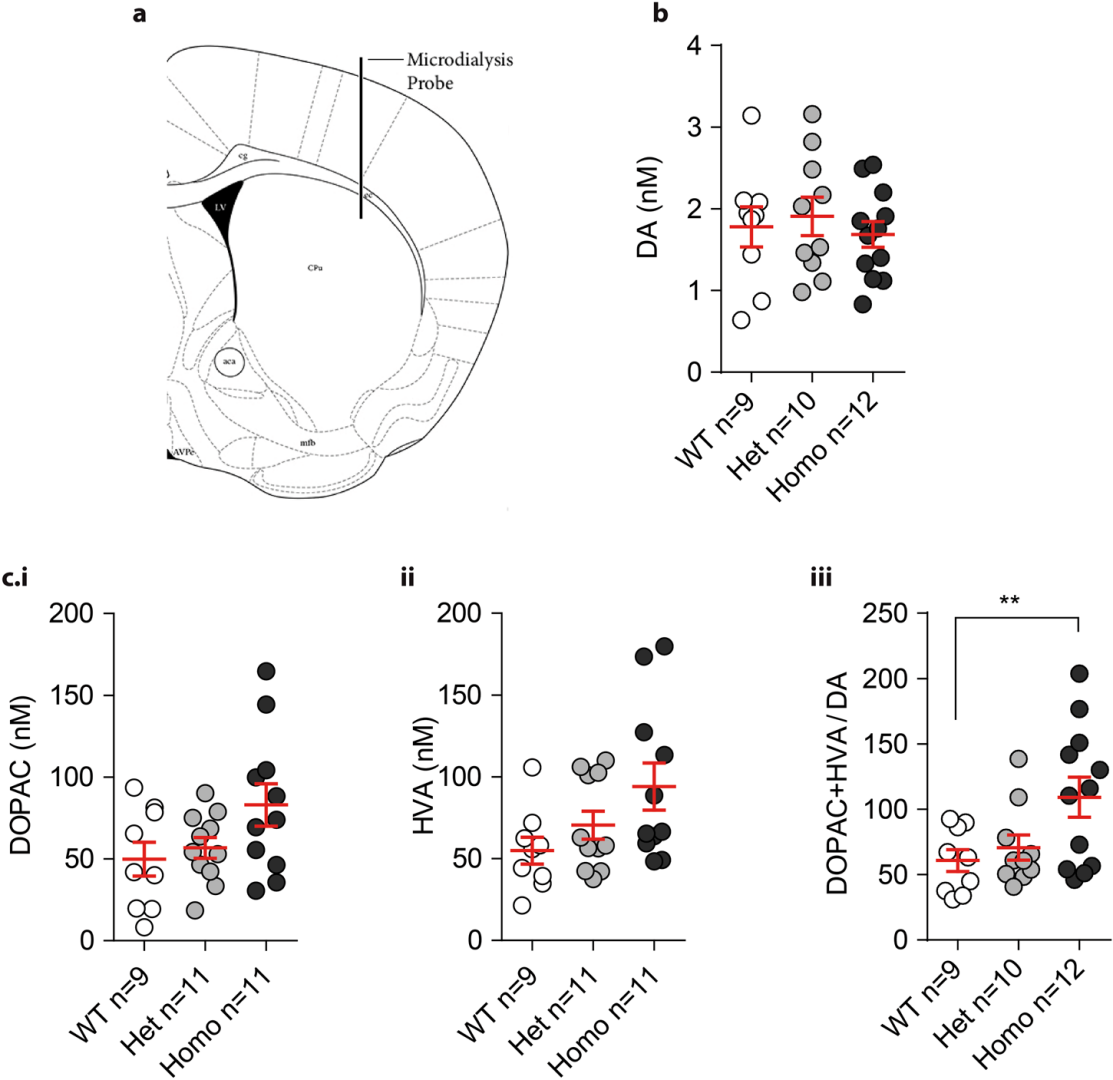
Extracellular striatal dopamine in VKI animals. **a** Placement of the probe (coordinates AP +0.5, ML +2.1, DV −2.8) for in-vivo detection of dopamine in the dorsolateral striatum. (Schematic adapted from Franklin, K.B.J. & Paxinos, G. The Mouse Brain in Stereotaxic Coordinates (copyright: Academic Press, an imprint of Elsevier, Amsterdam, 2013).^40^) **b** Average levels from four dialysates collected over an hour, every 15 min. No difference was found in overall basal levels of dopamine (1-way ANOVA *F*_2,28_ = 0.61, *p* *=* 0.54). **c** Dopamine metabolites total levels (DOPAC (i) and HVA (ii) and their ratio over levels of dopamine (iii). Homo VKI mice show similar, albeit towards increased, levels of DOPAC and HVA (i and ii, 1-way ANOVA *F*_2,28_ = 2.89, *p* = 0.07 and *F*_2,28_ = 3.06, *p* = 0.06, respectively), with a significantly higher ratio indicating greater turnover of dopamine (iii; 1-way ANOVA *F*_2,28_ = 6.44, *p* < 0.04; Bonferroni post-test WT vs Het *t*_(28)_ = 0.40, *p* = 0.99, WT vs Homo *t*_(28)_ = 3.20, *p* = 0.03)

Striata were rapidly dissected from VKI mice, tissue was homogenized and supernatant was assayed by HPLC for dopamine and its metabolites. We saw no genotype differences suggesting that the total production of dopamine and its metabolites are unaltered in VKI mice (Supp. Fig. 4a, b.i-iii, 1-way ANOVA *p* = 0.61, *p* = 0.58, *p* = 0.62, and *p* = 0.85, respectively). To evaluate dopamine neurotransmission in VKI mice, we measured extracellular levels in 3-month-old animals by in vivo microdialysis, as previous.^18^ Probes were inserted into the dorsolateral striatum and four samples, one every 15 min, were averaged (Fig. 5a). No difference in basal levels of dopamine was found in VKI mice compared to WT littermates (Fig. 5b; 1-way ANOVA *p* = 0.34). Dopamine metabolites, 3,4-dihydroxyphenylacetic acid (DOPAC) and homovanillic acid (HVA), were also not significantly elevated (Fig. 5c.i,ii; 1-way ANOVA *p* = 0.07 and *p* = 0.06, respectively). However, the ratio of DOPAC and HVA metabolites to dopamine was significantly elevated (Fig. 5c.iii; 1-way ANOVA *p* < 0.05; Bonferroni post-test WT vs Het *p* = 0.99, WT vs Homo *p* < 0.05). Together, microdialysis results suggest increased dopamine turnover in the presence of the mutation.

### VKI mice show altered dopaminergic markers

**Fig. 6:**
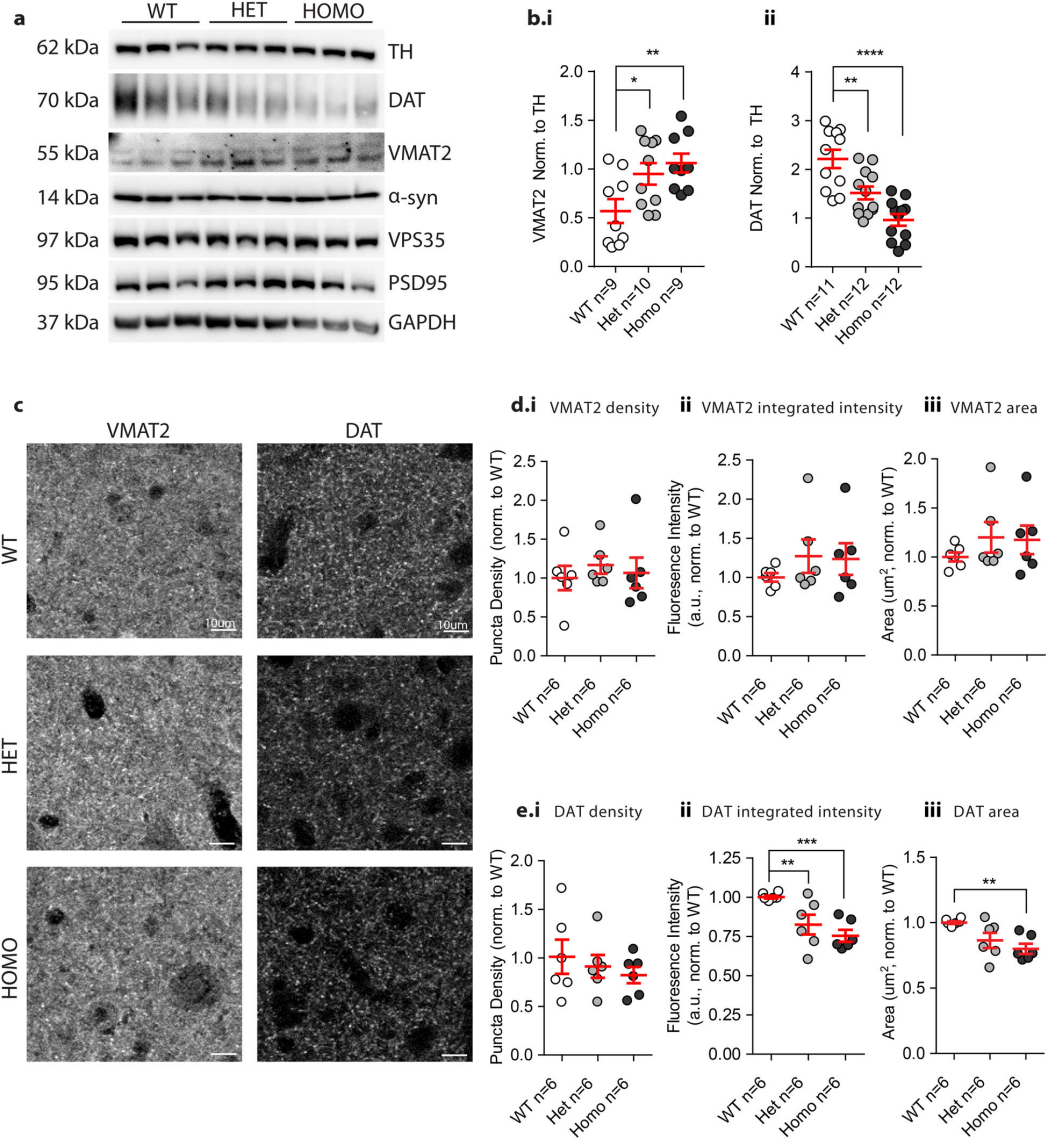
Dopaminergic markers DAT and VMAT2 in tissue and striatal slices from VKI mice. **a** Representative western blots of dopaminergic terminal markers VMAT2 and DAT in VKI mice. Blots are cropped; for full blots see Supp. Fig. 2. Samples on each blot derive from the same experiment and were processed in parallel. **b** Densitometry analysis was conducted by normalizing VMAT2 and DAT intensity to TH loading control. In VKI mice, VMAT2 levels were significantly increased (**b**.i, 1-way ANOVA *F*_2,25_ = 5.30, *p* < 0.01, Bonferroni post-test WT vs Het *t*_(25)_ = 2.45, *p* = 0.042, WT vs Homo *t*_(25)_ *=* 3.09, *p* < 0.01), with a significant decrease in total DAT protein levels (**b**.ii, 1-way ANOVA *F*_2,32_ = 17.75, *p* < 0.0001, Bonferroni post-test WT vs Het *t*_(32)_ = 3.32, *p* < 0.01, WT vs Homo *t*_(32)_ = 5.95, *p* < 0.0001). **c** Representative confocal images of VMAT2 (left) and DAT (right) in the dorsolateral striatum of 3-month-old VKI mice. **d** IHC analysis of VMAT2 shows no changes in VMAT2 puncta density (**d**.i, 1-way ANOVA *F*_2,15_ = 0.2793, *p* = 0.76), integrated intensity (**d**.ii, 1-way ANOVA, *F*_2,15_ = 0.726, *p* = 0.50), and area (**d**.iii, 1-way ANOVA, *F*_2,15_ *=* 0.745, *p* = 0.49) in VKI compared to WT littermates. **e** DAT puncta density was unchanged (**e**.i, 1-way ANOVA *F*_2,15_ = 0.518, *p* = 0.61), while integrated intensity (**e**.ii, 1-way ANOVA, *F*_2,15_ = 8.81 *p* < 0.01, Bonferroni post-test WT vs Het *p* < 0.05 *t*_(15)_ = 2.90, WT vs Homo *t*_(15)_ = 4.07, *p* < 0.01) and area were significantly decreased (**e**.iii, 1-way ANOVA, *F*_2,15_ = 6.36, *p* < 0.001, Bonferroni post-test WT vs Homo *t*_(15)_ = 3.45, *p* < 0.01). For VMAT2 and DAT immunohistochemistry “*n*” is the average value of all slices per animal

To explore these alterations in dopamine release, we sought to investigate the synaptic machinery responsible for the packaging and re-uptake of dopamine. Compared to WT littermates, VKI mice displayed a significant increase in VMAT2 protein levels (Fig. 6a,b.i, 1-way ANOVA *p* < 0.01, Bonferroni post-test WT vs Het *p* < 0.05, WT vs Homo *p* < 0.01). Conversely, a notable loss of total DAT protein (Fig. 6a,b.ii, 1-way ANOVA *p* < 0.0001, Bonferroni post-test WT vs Het *p* < 0.005, WT vs Homo *p* < 0.0001) was observed. Results were normalized to TH for tissue specificity but remain consistent when normalized to GAPDH (Supp. Fig. 6c.i, ii). These changes occurred independently of any differences in post-synaptic scaffold protein 95 (PSD95; Supp. Fig. 5c; 1-way ANOVA *p* = 0.65; Supp Fig. 6c.iii) suggesting *Vps35* p.D620N expression does not globally disrupt synaptic connections in the striatum.

To expand on these results, we employed fluorescence immunohistochemistry and confocal imaging in the dorsolateral striatum using antibodies against VMAT2 and DAT. No difference was observed between genotypes, shown by animal, when analyzed for VMAT2 positive puncta density (Fig. 6c,d.i; 1-way ANOVA *p* = 0.76), puncta integrated intensity (Fig. 6c,d.ii; 1-way ANOVA *p* = 0.50) or puncta area (Fig. 6c,d.iii; 1-way ANOVA *p* = 0.49) relative to WT littermates. Consistent with western blotting, VKI mice displayed a significant decrease in DAT puncta integrated intensity (Fig. 6c,e.ii; 1-way ANOVA *p* < 0.01 Bonferroni post-test WT vs Het *p* < 0.05, WT vs Homo *p* < 0.01), and in puncta area (Fig. 6c,e.iii; 1-way ANOVA *p* < 0.001 Bonferroni post-test WT vs Homo *p* < 0.01) within the dorsal striatum. However, VKI mice displayed no change in DAT puncta density (Fig. 6c,e.i; 1-way ANOVA *p* = 0.61), which along with VMAT2 puncta density suggests no observable loss of nigrostriatal synaptic release sites. This claim is supported by analysis of the pre- and post-synaptic proteins, synapsin1 and PSD95, whose densities and colocalization remain unchanged (Supp. Fig. 5b.i, ii, iii; Synapsin1 density ANOVA *p* = 0.37, PSD95 density *p* = 0.33, and synapsin1/PSD95 colocalization *p* = 0.57, respectively). Immunohistochemical analyses of VMAT2 and DAT, normalized and compared to WT controls, are also shown by slice (Supp. Fig. 5d,e).

To evaluate whether alterations were specific to dopaminergic neurons, we measured total levels of the serotonin transporter (SERT) and the norepinephrine transporter (NET) by western blotting. The mean striatal levels of SERT were significantly different (1-way ANOVA; *p* < 0.03), however multiple comparisons testing failed to determine differences between individual genotypes (Supp. Fig. 6a,b.i; Bonferroni post-test *p*>0.05). In contrast, levels of NET and D2 were unchanged in VKI animals when compared to WT littermates (Supp. Fig. 6b.ii; 1-way ANOVA; *p*=0.16 and Supp. Fig. 7a.i-ii; 1-way ANOVA *p* = 0.56, respectively). Lastly, given the role of VPS35 in α-synuclein processing^19,20^ we evaluated α-synuclein levels. Immunohistochemical analysis of slices from VKI brains show no differences in α-synuclein puncta density or distribution in the SNpc (Supp. Fig. 8a.ii, 1-way ANOVA *p* = 0.72), nor in protein levels quantified by western blotting of striatal tissue (Supp. Fig. 8b, 1-way ANOVA *p* = 0.75).

## Discussion

At 3 months of age, VKI mice appear normal with regard to *Vps35* expression levels, breeding, survival and weight (Fig. 1 and Supp. Fig. 1). They exhibit no gross motor dysfunction (Fig. 2) or loss of striatal TH-immunopositive dopaminergic innervation, and no loss of nigral neurons (Fig. 3). Given these observations, it was surprising to find increased capacity to evoke dopamine release in the dorsolateral striatum of VKI slices (Fig. 4). Heterozygous and homozygous animals also show an increase in the decay times of evoked dopamine release, consistent with elevated extracellular dopamine and impaired re-uptake (Fig. 4 and Supp. Fig. 3f). Total D2 receptors levels are similar between genotypes (Supp. Fig. 7). Nevertheless, the inhibitory effect of quinpirole suggests D2 signaling is augmented in VKI slices (although comparable inhibition was observed for all genotypes by ~30 min) (Fig. 4 and Supp. Fig. 3e).

Basal levels of extracellular dopamine appear normal by in-vivo microdialysis but the ratio of DOPAC and HVA to dopamine was elevated in homozygous VKI (Fig. 5). We surmise higher levels of dopamine, initially released, are more rapidly broken-down. However, further assessment of the level and enzymatic activity of MAO and COMT in striatal tissue may prove informative. Although the temporal resolution of FSCV and microdialysis are different, our findings for both techniques are parsimonious. In partial support, an independent line of *Vps35* p.D620N knock-in mice was recently published with normal motor function in similar behavioral paradigms, normal basal levels of dopamine and metabolites by in vivo microdialysis.^16^ However, depolarization-induced dopamine release, with 120 mM potassium chloride, was significantly decreased in homozygous animals compared to WT.^16^

In VKI striata DAT levels are significantly lower and VMAT2 levels are increased, as observed by western analyses, and validated by DAT immunohistochemistry (Fig. 6 and Supp. Fig. 6). Both transporters are essential for dopamine re-uptake and storage,^21^ and their differential expression has long been considered to contribute to the selective vulnerability of the SNpc in PD.^22,23^ In prior mouse models DAT overexpression is best described to cause midbrain cell loss but chronic reduction will also disrupt the balance of extra- to intracellular dopamine with consequent effects on signaling and homeostasis.^21,24^ In contrast, VMAT2-overexpressing mice have increased vesicular capacity to store dopamine and evoke release,^25^ and consequently are less vulnerable to methamphetamine-induced dopaminergic neurodegeneration.^26^ While complete VMAT2 deficiency is embryonically lethal, mice expressing low levels of VMAT2 have deficits in vesicular packaging that lead to dopamine depletion, with consequent behavioral alterations and α-synuclein aggregation.^21^ VKI mice now complement and extend this literature.

A correlation between elevated levels of VMAT2 and evoked dopamine release is observed, as commonly reported in recombination models,^25^ but intriguingly tissue dopamine levels are unchanged between genotypes. VKI littermates have a reduction of total DAT protein, correlating with increased decay time of evoked dopamine release, albeit incongruent with previous work that shows increased VMAT2 expression promotes the loss of DAT.^21^ Non-physiologic expression of VMAT2 may reduce DAT as a mechanism to limit re-uptake of released dopamine. In VKI mice, the changes in DAT may reflect deficits in activity-dependent transporter recycling to the plasma membrane via VPS35-positive endosomes.^27^ However, future work must examine whether VPS35 p.D620N directly affects retromer selection of DAT as a cargo.

Interestingly, mean protein levels of SERT, but not NET, were also increased in striata from VKI mice (Supp. Fig. 6a,b), although whether to compensate for dopaminergic dysfunction or due to a more direct mechanism is unclear and warrants further investigation using larger cohorts. Serotoninergic neurons can uptake dopamine through SERT, a phenomenon that is often linked to motor complications associated with PD treatment.^28^ While more work is needed to explore the serotoninergic and noradrenergic systems, retromer recycling of DAT may be particularly important in actively releasing dopaminergic terminals^29^ and in VPS35 parkinsonism. VMAT2 protein was significantly increased in VKI animals (Fig. 6a–d and Supp. Fig. 6c) but that excess may not be functional as dopamine levels in striatal tissue appear grossly normal (Supp. Fig. 4). Additional pharmacologic investigations of VMAT2 activity, with tetrabenazine or reserpine, might address this issue.^30^

VMAT2, DAT, and TH are all critical components in dopamine storage, release/reuptake and synthesis. Their activity is fine-tuned by dopamine D2/D3 autoreceptors, trace amine receptor signaling and molecular chaperones. Nevertheless, much of the coordination in these molecular pathways and processes remains enigmatic.^31^ VKI mice now provide a relatively physiological means to probe these relationships. For example, TH expression is not significantly different between genotypes but protein levels appear to be marginally increased in VKI mutant mice (Fig. 3b). Thus, further assessment of TH activity is warranted in the context of stimulated dopamine release.

Presynaptic dopaminergic phenotypes observed in VKI mice may require molecular and/or network compensation to mask organismal dysfunction, which may fail with age. Similar neurotransmission phenotypes have been reported in prior models based on mutations linked to late-onset parkinsonism.^18,32^ Many of these proteins affect the endosome recycling system and functionally converge about receptor recycling.^33^ For example, in human LRRK2 p.G2019S heterozygote carriers, initial elevations in serotonin and dopamine function decline with age, as symptoms manifest.^34,35^ This is similar to results garnered in G2019S knock-in mice.^17^ Notably, *Vps35*-deficiency is embryonic lethal, but in surviving neurons that deficiency impairs AMPA receptor trafficking, impedes dendritic spine maturation and decreases glutamatergic transmission.^13^ In contrast AMPA receptor cluster intensity is increased in human dopaminergic neurons derived from inducible pluripotent stem cells of VPS35 p.D620N heterozygotes.^11^ Such network effects involving excitatory and inhibitory neurotransmission may place excessive demands on synaptic proteostasis, ATP metabolism, mitochondria and autophagy. With time, chronic dysfunction may challenge compensatory systems, and lead to the insidious loss of dopaminergic neurons. In conclusion, further study of VKI mice may elucidate the ontology of VPS35 p.D620N parkinsonism. As VPS35 and LRRK2 impinge on the same biological pathway,^36–39^ VPS35 p.D620N heterozygotes may also inform the development of LRRK2 kinase inhibitors in genetically-defined and idiopathic PD.

## Materials and methods

### *Vps35* p.D620N knock-in mice and gene expression

Conditional and constitutive knock-in mice were generated by Ozgene PLC (Australia) using a gene targeting approach in C57Bl/6 embryo stem cells (Bruce4). Genetic engineering targeted the *Vps35* locus on murine chromosome 8 (85,260,392–85,299,802 reverse strand (GRCm38.p5); Ensemble genomic reference sequence ENSMUSG00000031696; NCBI mRNA reference sequence NM_022997.4). A floxed “mini-gene” consisting of: (1) splice acceptor, *Vps35* exon 15–17 coding sequence, a polyadenylation signal (pA), and; (2) a *PGK*-neomycin (neo)-pA selection cassette internally flanked by FRT sites, was inserted into intron 14. The 5′ targeting arm spanning endogenous exon 15 was used to introduce the *Vps35* g.85,263,520G>A (p.D620N) mutation. Conditional animals (denoted cVKI) have their endogenous gene (exons 1–14) spliced to the mini-gene cassette (exons 15–17, pA); the *PGK*-neo-pA selection cassette was removed by crossing to transgenic animals expressing FlpE-recombinase. Heterozygous cVKI mice were crossed to transgenic animals expressing Cre-recombinase to remove the floxed mini-gene cassette, enabling heterozygous constitutive *Vps35* p.D620N expression (denoted VKI mice; Fig. 1). Removal of the Cre transgene was achieved by selective breeding to C57Bl/6J stock in subsequent generations. Genotyping to ensure the absence of neo, FlpE- and Cre-recombinase transgenes, and to verify the presence of the *Vps35* g.85,263,520G>A (p.D620N) mutation, was performed using a TaqMan® allelic discrimination assay on a 7900HT System (Applied Biosystems). Intron 14–exon 15 of the modified genomic locus in homozygous VKI animals was ultimately confirmed by Sanger sequencing, using ABI 3730xls instrumentation (Applied Biosystems). The VKI strain has been deposited in Jackson Labs (www.jax.org; *Vps35* knock-in: B6(Cg)-Vps35tm1.1Mjff/J; Stock No: 023409) with open distribution supported by the Michael J Fox Foundation.

All breeding, housing, and experimental procedures were performed according to Canadian Council on Animal Care regulations with appropriate ethical approvals (UBC RISe Ethics approvals A15-0105 & A16-0088; PI Matt Farrer). VKI mice were maintained for >5–10 generations, kept on a reverse cycle (light on from 7pm to 7am) and single-sex group-housed in enrichment cages after weaning at post-natal day 21. For all experiments, 3 months old male animals were used. Post-mortem ear notches were taken for post-hoc genotyping. All experiments, data processing and analysis was conducted in a blinded manner.

### mRNA expression

Mice were sacrificed by rapid decapitation and brain tissue was microdissected (<6 min). To quantify *Vps35* expression, cerebellar RNA was isolated using RNeasy Kit (Qiagen) and reverse transcribed using High Capacity cDNA Kit (Applied Biosystems), according to the manufacturer’s instructions. The reactions were incubated in a thermal cycler for 10 min at 25°C, 120 min at 37°C, 5 min at 85°C, and then held at 4°C. To determine the relative expression of *Vps35*, we amplified by means of Real-time PCR using the 7900HT System with SYBR® Green PCR Master Mix (Applied Biosystems) at 95°C for 10 min followed by 40 cycles of 15s at 95°C and 1 min at 57°C. The relative expression of mutant *Vps35* was analyzed with the ΔΔCt method using the geometric mean of endogenous control genes, *Actb*, *Gapdh*, and *Rpl19*. Primers sequences are available in supplemental data. The fold change (2^−ΔΔCt^) expression was normalized to the expression of WT littermates.

### Behavioral testing

Mice undergoing experimental procedures were weighed prior to testing. All tests were performed in the morning between 8:30am and 11:30am. All animals were assessed at 3 months of age, and all experimentation and analysis was conducted with the experimenter blinded to genotype. After 3-day familiarization to experimenter handling, mice underwent the following paradigms. *Open field (OF)*: mice explored an open arena (48cm×48cm) for 15 min, as previous.^17^ Videos were analyzed post-hoc with ANY-maze behavioral tracking software (Stoelting). *Cylinder*: mice were placed in a 1L beaker and recorded for 5 min as previous.^17^ The number of rearings and forelimb wall contacts was scored manually offline. *Rotarod*: Motor performance was evaluated using an accelerating rotarod (Model 7650, Ugo Basile). Mice were recorded during a training session to establish baseline falling latency, and in test sessions. Training consisted of six trials over 2 days. For each trial the rotation was set at a constant speed, but higher from trial to trial, so all mice have the ability to run at 22 revolutions per minute (RPM). During the test session, the rod was left accelerating from 4 to 40RPM over 5 min. Fails were scored if mice: (1) fell from the device, or (2) gripped the rod making a passive revolution. Videos were analyzed offline and number of fails and latency to first fail were scored.

### Protein analysis

For western blot (WB) analysis, dissected striatal brain tissue was lysed in HEPES buffer (20 mM HEPES, pH 7.2, 50mM KAc, 200 mM Sorbitol, 2 mM EDTA, 0.1% Triton X-100, 0.5% NP-40) containing protease and phosphatase inhibitors (Roche), homogenized, and incubated (on ice, 45 min, gentle agitation every 15 min). Lysates were cleared by centrifugation at 4000×*g* for 12 min at 4 °C and supernatant was quantified by BCA assay (Pierce). Lysates were denatured in 1× LDS sample buffer (Thermo Fisher Scientific) and heated for 10 min at 70 °C and 10–15 µg of protein was resolved by SDS-PAGE as previously described,^19^ or using NuPage 4–12% Bis–Tris gel (Thermo Fisher Scientific) and transferred to PVDF membranes (EMD Millipore). Membranes blocked with 5% nonfat milk in Tris-buffered saline containing 0.1% Tween 20 (TBST) were probed overnight at 4 °C with primary antibodies diluted in 3% BSA (Sigma-Aldrich) in PBS. The following day, membranes were washed in TBST (3 × 5 min) at room temperature (RT) followed by incubation for 1 h at RT with horseradish peroxidase-conjugated anti-mouse, rabbit, or Rat IgG (Santa-Cruz Biotechnology). Blots were washed in TBST (3 × 5 min) at RT, and developed using enhanced chemiluminescence plus reagent (Thermo Scientific). Subsequent imaging was performed with a Chemi-Doc imaging system (Cell-Bio), and images were analyzed for band intensity with Image J software. The following primary antibodies were used for WB: Mouse anti-VPS35 (Abnova, H00055737-M02); anti-GAPDH (Cell Signaling, 2118); anti-PSD95 (6G6-1C9, Thermofisher Scientific, MA1-045); anti-TH (Sigma-Aldrich, T2928); Rabbit anti-mouse VMAT2 (Phoenix Pharmaceuticals, Inc), Rat anti-DAT (EMD Millipore, MAB369), Rabbit anti-SERT (Synaptic Systems, 340 003), Rabbit anti-NET (Synaptic Systems, 260 003), and Rabbit anti-D2 receptor (Millipore AB5084P). The detection of primary antibodies was achieved by probing membranes with Donkey anti-Rabbit or mouse IgG conjugated horseradish peroxidase (HRP) (Santa-Cruz Biotechnology, sc-2318, sc-2313, respectively), or Rabbit anti-Rat IgG HRP (Abcam, ab6734).

### Immunohistochemistry

Three-month-old mice were terminally anesthetized (sodium pentobarbital 240 mg/kg, i.p.) and intracardially perfused with PBS then 4% paraformaldehyde (PFA). Brains were extracted, post-fixed overnight (4% PFA, 4 °C) then cryoprotected with increasing sucrose gradient (10%, 20%, 30% in PBS O/N, 4 °C). Coronal slices (30 µm) were obtained by cryostat. Where necessary, antigen retrieval using 10 mM sodium-citrate plus 0.05% Tween (pH 6; 30 min, 37 °C) was performed. Sections were rinsed with 0.1% Triton-X in 1x PBS (PBST; 3 × 10 min), blocked in 3% milk in 0.03% PBST (30 min, RT) followed by a second block in 10% normal goat serum (NGS) in 0.03% PBST (30 min, RT). Primary antibodies: rabbit anti-synapsin1 (AB1543P, Millipore; 1:500), anti-VMAT2 (H-V008, Phoenix Pharmaceuticals; 1:500), mouse anti-PSD95 (MA-045, Thermo Scientific; 1:250), chicken anti-Tyrosine Hydroxylase (TH; ab76442 Abcam; 1:1000), goat anti-VPS35 (S-18, sc-55805, Santa Cruz; 1:250) and Rat anti-DAT (N-terminal, MAB369, Millipore; 1:500) were applied in 5% NGS + 0.01% NaN_3_ in 0.01% PBST; overnight 4 °C prior to washing (3 × 10 min PBST) and secondary incubation with species specific Alexafluor IgG secondary antibodies (90 min RT, Invitrogen; 1:1000). Tissues were washed again in 0.1% PBST (3 × 10 min), then mounted using DAPI Fluoromount-G® (0100–20, SouthernBiotech). Images were acquired using a 60x oil objective on an Olympus Fluoview FV-1000 confocal laser scanning microscope (9 × 0.33 μm step size). Images were then stacked (three stacks of 3 individual images viewing 1 μm tissue depths) and masked using FIJI ImageJ software (NIH, USA) and analyzed using Cell Profiler image analysis software (v.2.1.1).

### Stereological cell counts

For slices expressing TH-positive labeling in the SNpc, optical fractionator sampling was conducted on images acquired by confocal laser-scanning microscope (Olympus Fluoview FV1000, 60× oil) and whole-volume estimates produced from tiled images acquired on an EVOS FL microscope (Thermo Fisher Scientific), as described by stereology.info (MBF Bioscience). Dopaminergic neurons of the SNpc were identified by TH-positive immunolabeling.^40^ For our counting to encompass the full rostro-caudal extent of the relevant SNpc dopamine nuclei, a section-sampling fraction of 1/3 was used to analyze a total of ~10 sections for each brain. Average slice thickness was estimated expecting minimal shrinkage as the sections were parallel processed and mounted in aqueous medium. A guard height of 2 μm was used with a counting frame height of 28 μm. The counting frame size measured 159 μm width (*x*) by 159 μm height (*y*), which was chosen to include ~10–20 neurons per frame. The position within the SNpc was roughly maintained across all sections in all animals sampled. The area-sampling fraction was determined by the counting frame area/SNpc area, as measured by tiled images and area measuring software on the EVOS FL microscope.

### Fast-scan cyclic voltammetry

FSCV was conducted in the dorsolateral striatum in 300 μm thick coronal slices from 3 months old male mice, as previous.^17^ Slices were perfused at RT with artificial cerebrospinal fluid [ACSF—containing in mM: 130 NaCl, 10 glucose, 26 NaHCO_3_, 3 KCl, 1 MgCl_2_·6H_2_0, 1.25 NaH_2_PO_4_ (monobasic monohydrate), 2 CaCl_2_; pH 7.2–7.4, mOsm 290–310] and oxygenated (95% O_2_, 5% CO_2_) at room temperature for >1 h prior to experiments. Individual slices were then placed in a recording chamber at temperature 22–26 °C, and perfused at 1–2 mL/min with ACSF. Stimuli (150 μs duration) were delivered by nickel-chromium bipolar electrodes (made in house) placed in the dorsolateral striatum, optically isolated (A365, World Precision Instruments, USA) and controlled/sequenced with ClampEx software. Voltammetric responses were recorded, standardized and analyzed with an Invilog Voltammetry system and software (Invilog Research Ltd., Finland). Carbon fiber electrodes (diameter: 32 μm, length: 300 μm, sensitivity: >20 nA/μM) were purchased prefabricated (Invilog) and placed within 100–200 μm of the stimulating electrode in the dorsolateral striatum (as shown in Supp. Fig. 3). Triangular waveforms (ramp from −400 mV to 1200 mV to −400 mV, 10 ms duration at 10 Hz) were used to detect the oxidation and redox peaks for dopamine between 700 and 800 mV (see voltammogram in Fig. 4). Striatal field excitatory postsynaptic potential (fEPSPs) was also recorded to ensure slice viability during the duration of each experiment. Input/Output paradigms consisted of increasing single pulse stimuli (100–700 μA, delivered every 2 min/0.0083 Hz) to determine ~70% maximum response used for the remainder of the experiment. Three single pulses were delivered at 0.0083 Hz to assess baseline stability, followed by a single paired-pulse stimulus (4 s IPI). A train of stimuli (100 Hz) was applied for 1 s to evaluate the maximal response at the given intensity. During drug wash-in, single pulse stimulations were continued at 0.0083 Hz for 10 min. A repeat of the 3 single simulations, paired-pulses and train were then recorded in the drug condition. At the end of each recording session, a three-point calibration of each carbon fiber electrode was conducted (final concentrations 0.1, 0.5, 1.0 μM dopamine in ACSF). Quinpirole [(−)-Quinpirole hydrochloride, Tocris Biosciences] was employed to assay pre-synaptic D2-receptor agonism at a concentration (50 nM), previously shown to reduce dopamine response by 20–50%.^17^

### Microdialysis

Under isoflurane anesthesia, a CMA 7 microdialysis probe (1 mm dialyzing membrane; Harvard Apparatus) was lowered into the mouse striatum according to the following coordinates: AP +0.5, ML +2.1 from bregma, DV −2.8 below dura, as previous.^18^ The probe was secured to the skull by glass ionomer cement (Instech Laboratories) and a stainless steel screw. During surgery, animals were given lidocaine prior to incision (30 µL, s.q.), meloxicam (1 mg/kg, s.q.) and warm 0.9% saline (s.q.) at the end of the procedure. Following surgery, mice were allowed to recover and experiments were run 24 h after probe implantation. Microdialysis probes were perfused at a flow rate of 1.5 µL/min with a modified Ringer’s solution (in mM: 147 NaCl, 3 KCl, 1.2 MgCl_2_·6H_2_O and 1.2 CaCl_2_). Samples were collected every 15 min, starting 2 h after the onset of probe perfusion, into vials containing 2 µL of 10 mM acetic acid.

Dopamine and its metabolites (DOPAC and HVA) were measured by HPLC coupled to electrochemical detection (ALEXYS Neurotransmitter platform, Antec). Five microliters of sample were automatically injected (AS 110 Autosampler, Antec) onto an Acquity UHPLC BEH C18 analytical column (1 mm inner diameter, 10 cm length; Waters) perfused at a flow rate of 50 µL/min (LC 110S pump, Antec) with a mobile phase containing 100 mM phosphoric acid, 100 mM citric acid, 0.1 mM EDTA, 600 mg/l octan sulfonic acid sodium salt and 8% acetonitrile (pH 3). Dopamine, DOPAC and HVA were detected by means of an electrochemical detector (Decade II, Antec) with cell potential set at 0.8 V vs salt bridge. Retention times were: 2.57 ± 0.2 min for DOPAC, 3.82 ± 0.2 min for dopamine and 5.18 ± 0.2 min for HVA. Mice were euthanized after the experiment and brains collected to confirm probe placement by immunohistochemistry.

### Striatal tissue dopamine quantification by HPLC

Striatal tissue was mechanically homogenized in a solution of 100 uM EDTA and 0.1 mM perchloric acid. Samples were cleared by centrifugation at 14,000×*g* for 10 min at 4 °C and supernatant separated from pellet, then dopamine and its metabolites were measured by HPLC as above. The pellet was lysed in HEPES buffer (20 mM HEPES, pH 7.2, 50 mM KAc, 200 mM Sorbitol, 2 mM EDTA, 0.1% Triton X-100, 0.5% NP-40) containing protease and phosphatase inhibitors (Roche), homogenized, and incubated (on ice, 45 min, gentle agitation every 15 min). Lysates were cleared by centrifugation at 4000×*g* for 12 min at 4 °C and supernatant was quantified by BCA assay (Pierce), to normalize monoamines levels to protein concentration.

### Statistics and data reporting

Data are presented throughout as mean ± SEM where “*n*” equals the number of animals, or else are shown as per slice “*n*” for the total number of animals (indicated in parentheses). Sample sizes estimates used the resource equation method^41^ but were refined using G power software^42^ with pilot data (*n* = 3) on effect size (mean/standard deviation within groups) for 1- *beta* = 0.8 and *alpha* = 0.05. Throughout the study, comparisons were conducted by 1- or 2-way ANOVA with appropriate post-hoc tests, as detailed in the text, using Prism 6.0 (GraphPad, San Diego, CA, USA).

## Electronic supplementary material

Supplementary figures and legends

## Data Availability

The authors declare that the data supporting the findings of this study are available within the paper and its supplementary information files. Additional, raw values may be obtained from the corresponding author, Prof. Matt Farrer, upon request.

## Electronic supplementary material

**Supplementary information** accompanies the paper on the *npj Parkinson’s Disease* website (10.1038/s41531-018-0063-3).
