# Supplementary material for: Altered dopamine release and monoamine transporters in Vps35 p.D620N knock-in mice
Source: NPJ Parkinsons Dis. 2018 Aug 21;4:27. doi: 10.1038/s41531-018-0063-3 (PMC6104078; doi:10.1038/s41531-018-0063-3)
Supplement: Supplementary file 1 — Supplementary figures and legends [file 41531_2018_63_MOESM1_ESM.docx]

**Altered dopamine release and monoamine transporters in Vps35 p.D620N knock-in mice**

Stefano Cataldi^1^*, Jordan Follett^1^*, Jesse D. Fox^1^, Igor Tatarnikov^1^, Chelsie Kadgien^1^, Emil K. Gustavsson^1,2^, Jaskaran Khinda^1^, Austen J. Milnerwood^1$^ & Matthew J. Farrer^1$^

**Supp.Fig.1 VKI mice weight at 3 months of age**


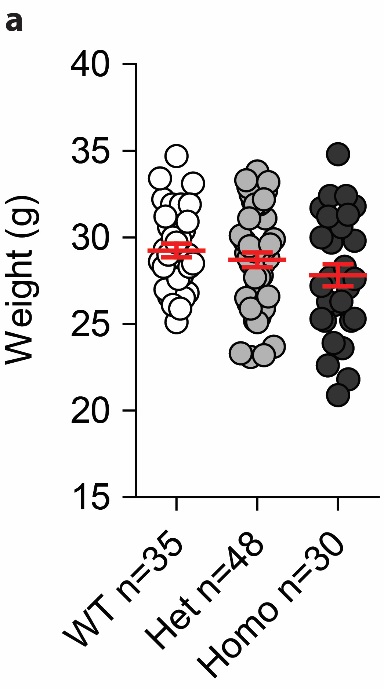


**a**) Weight for VKI and WT littermate mice were comparable (1-way ANOVA F_2,18_ = 2.17, *p*=0.76).

**Supp.Fig.2 Unprocessed western blots shown in Fig.6a.**


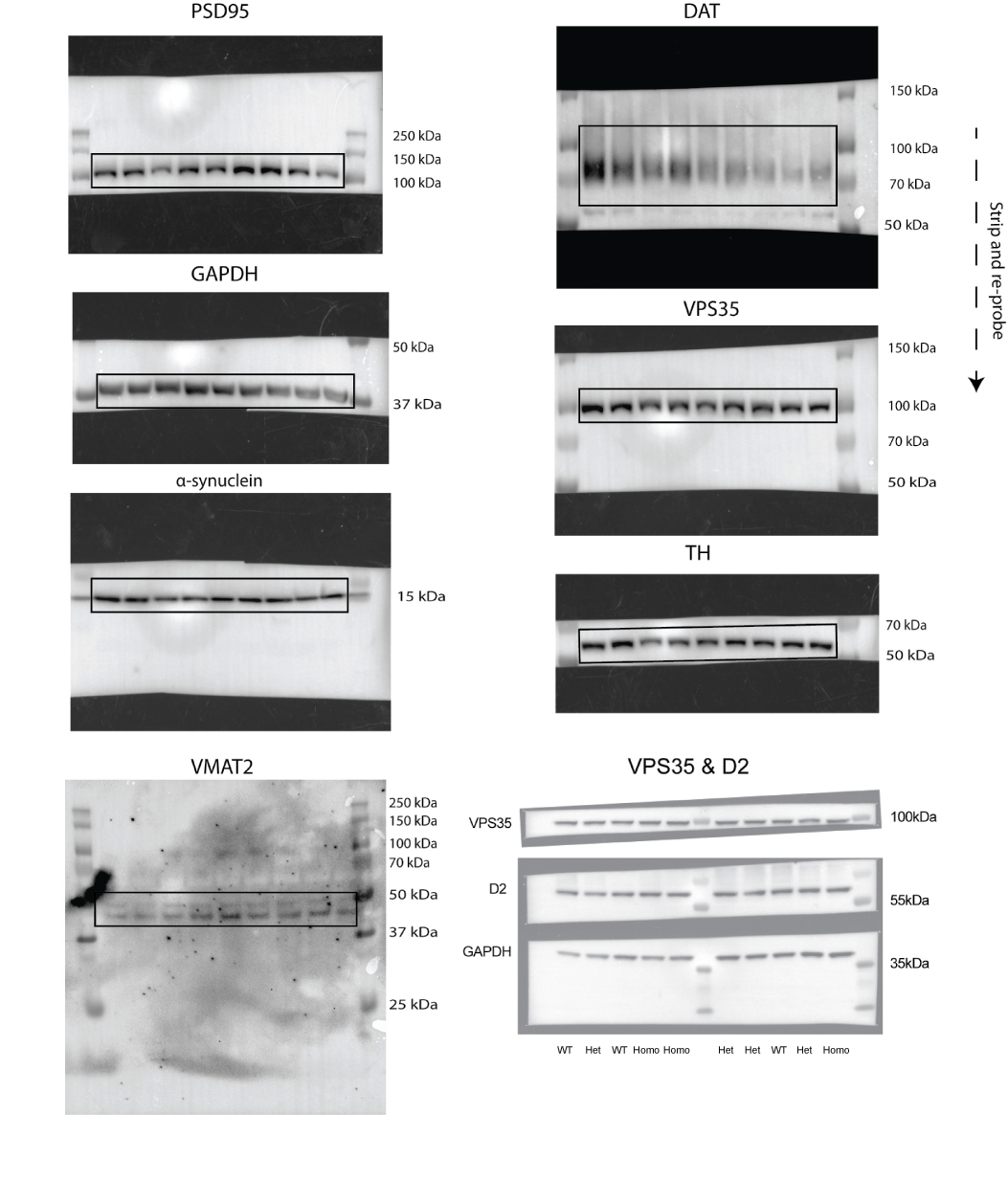


Uncropped blots merged with corresponding ladder channel. Images were split into individual channels for presentation in Figure 6 and Supp.Fig.5, 7 & 8. Black boxes mark where western blots have been cropped for presentation, or else each lane is labelled for comparison (VPS35 & D2 blot, bottom right). Molecular weights and antibody specificity are as indicated. Samples in all blots derive from the same experiment and were processed in parallel.

**Supp.Fig.3a-d Additional evaluation of evoked dopamine in VKI brain slices.**


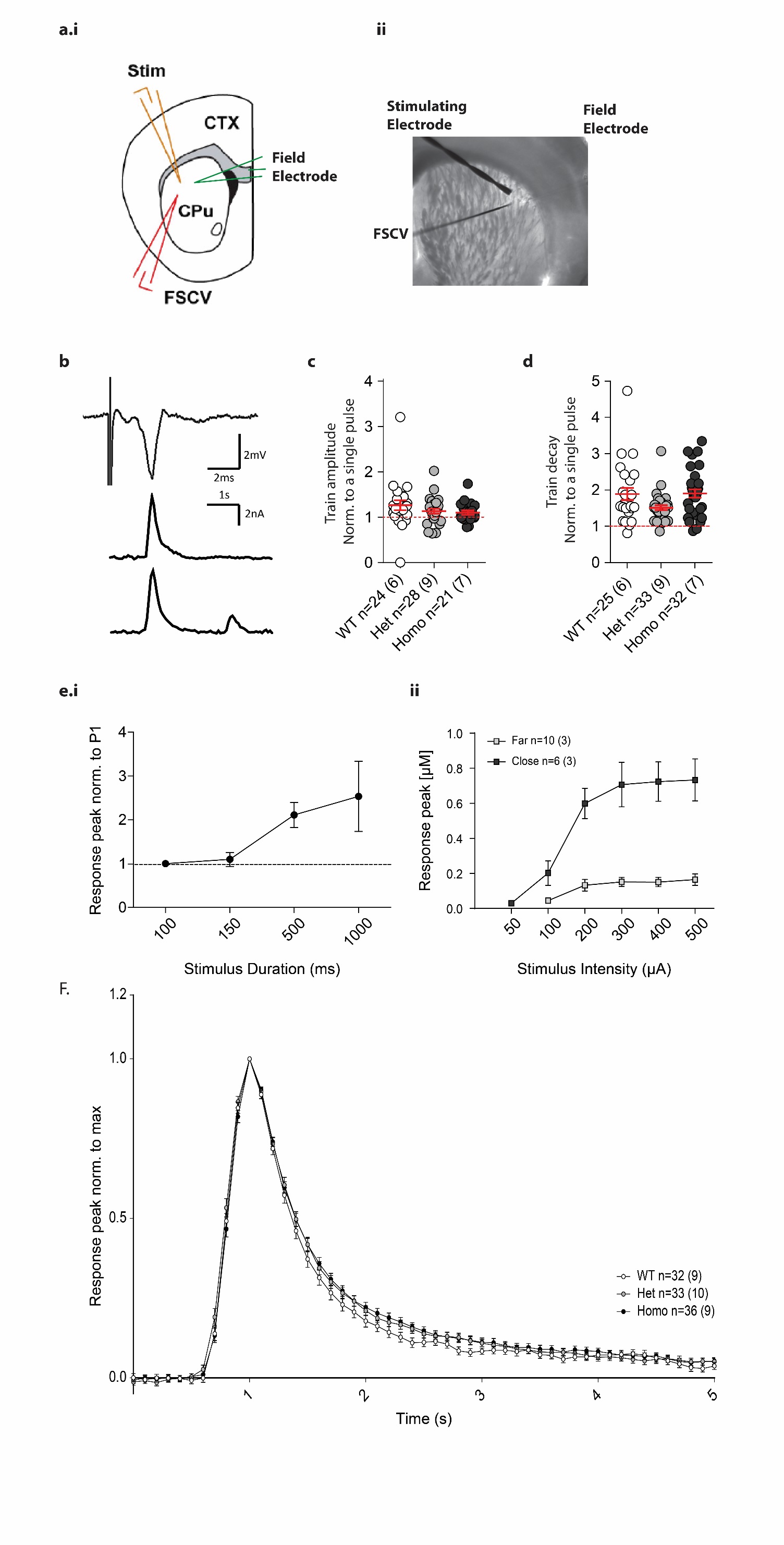


**a.i**) Schematic adapted from Franklin, K.B.J. & Paxinos, G. The mouse brain in stereotaxic coordinates (copyright: Academic Press, an imprint of Elsevier, Amsterdam, 2013) ^40^ (**a.ii**) image of the placement of stimulating (Stim) electrode and carbon-fiber microelectrodes (FSCV) in the dorsolateral striatum of *ex-vivo* brain slices. **b**) Examples of a single pulse field response (top), and concomitant dopamine response (middle), and dopamine paired-pulse (4s IPI) recordings (bottom). Example signals correspond to an average peak at 50-70% of maximal stimulation. **c**) WT and VKI slices show comparable dopamine release in response to a train of stimulus (100Hz, 1s) presented as train amplitude normalized to that of a single pulse (1-way ANOVA *F*_2,70_ = 1.28, *p*=0.82). **d**) Train decay time normalized to that of a single pulse was greater for WT and Homo slices (1-way ANOVA *F*_2,87_ = 3.946, *p*<0.05, the data does not reach significance with post-hoc test). Because not all animals were tested for the train paradigm, the number of slices is lower for **c** & **d**, compared to other paradigms.

**Supp.Fig.3e-f Additional evaluation of evoked dopamine in VKI brain slices.**


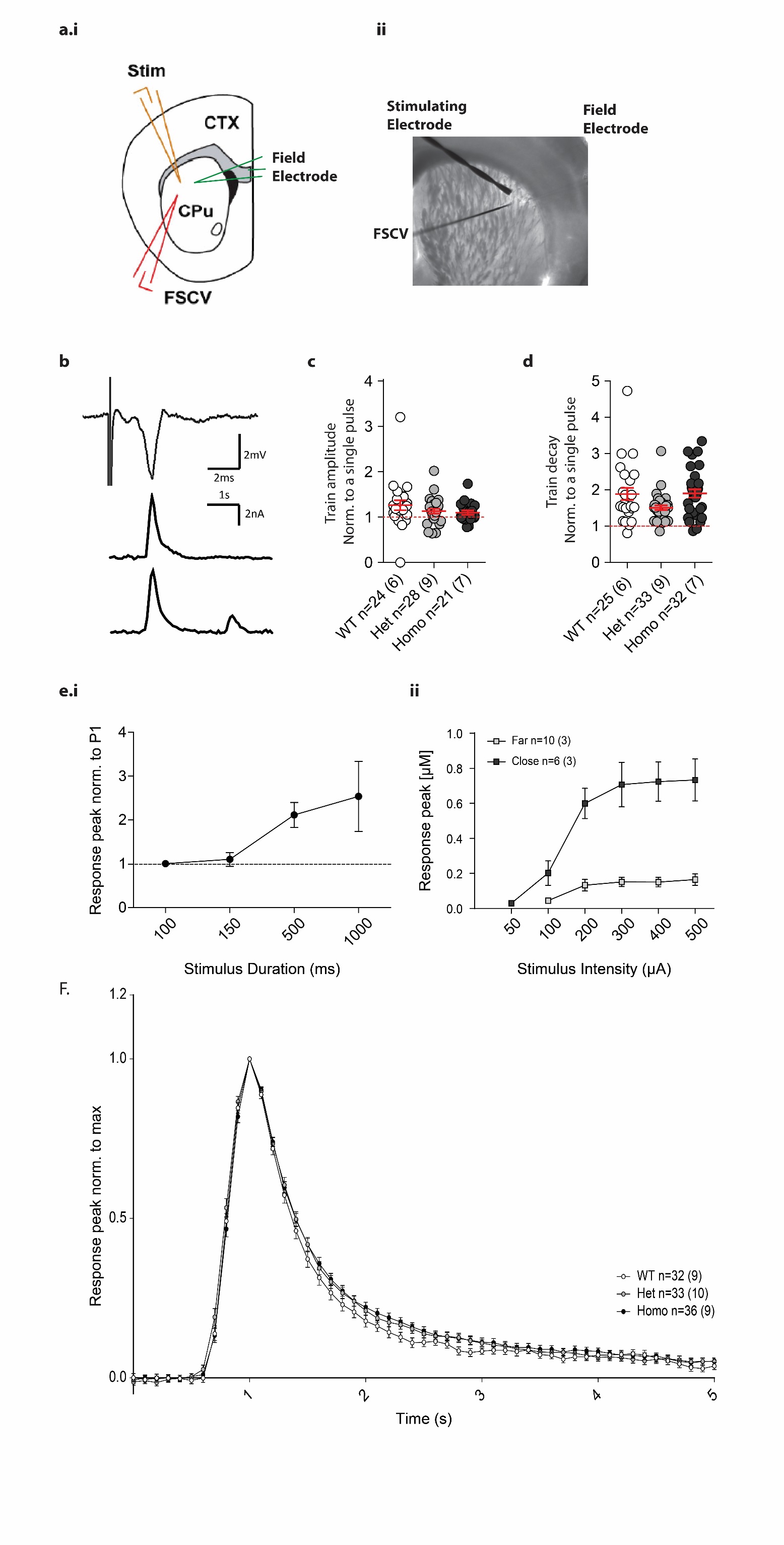


**f**

**e**) The effect of (i) stimulus duration and (ii) distance between the electrodes on evoked dopamine release. Increasing the duration from 150 µs to 500+ µs doubled the amplitude (n=4 animals). Distance between the carbon fiber recording electrode and the stimulating electrode had a pronounced effect on the concentration of evoked dopamine (far ~200 µm n=10 (3), close ~100 µm n=6(3)). **f**) Peak-normalized transformation of the data presented in Fig.4**a**. A modest slowing is maintained in this presentation of averaged trace data demonstrating the effect of genotype upon decay times, consistent with significantly increased decay tau observed in slices from heterozygous and homozygous mutants (Fig.4**d**).

**Supp.Fig.4 Evaluation of total levels of dopamine and metabolites in striatal tissue from VKI animals**

**
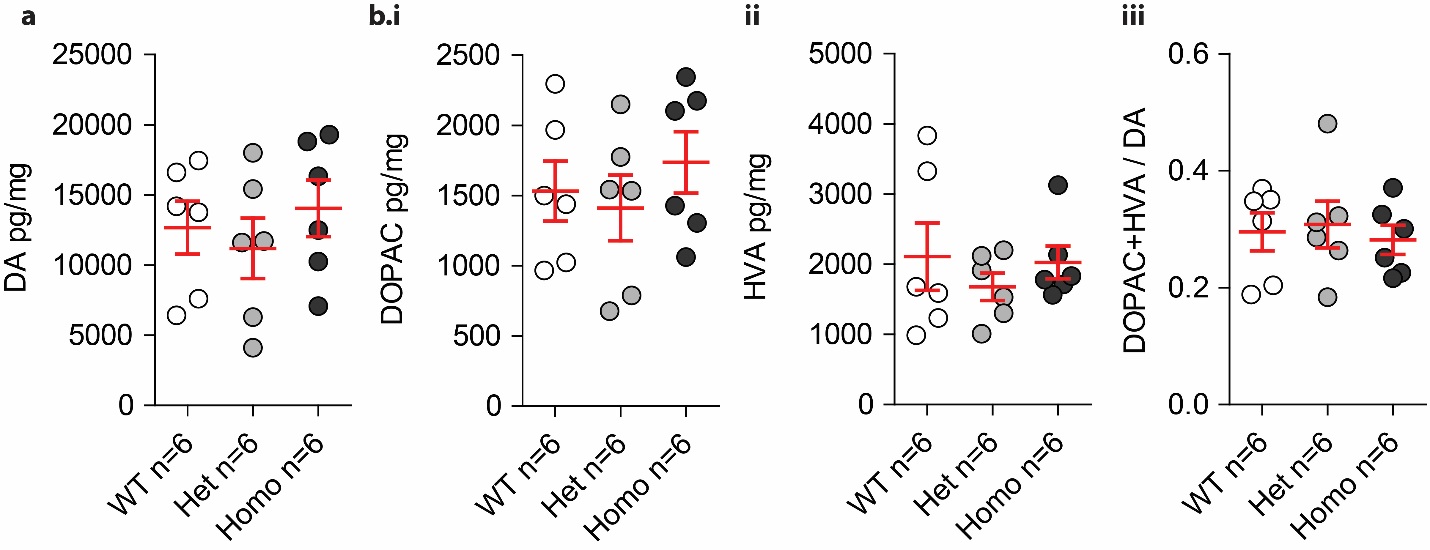
**

Striatal tissue was processed for monoamine detection by HPLC. When compared to WT mice, VKI animals showed no differences in total levels of dopamine (**a**, 1-way *F*_2,15_ = 0.5, *p* = 0.61) or metabolites (**b**.i & .ii, 1-way ANOVA *F*_2,15_ = 0.54, *p* = 0.58, & *F*_2,15_ = 0.48, *p* = 0.62, for DOPAC and HVA respectively), nor their ratio (**b**.iii, 1-way ANOVA *F*_2,15_ = 0.15, *p* = 0.85).

**Supp.Fig.5a-c. Synaptic markers synapsin1 & PSD95 in striatal tissue and slices from VKI mice.**

**
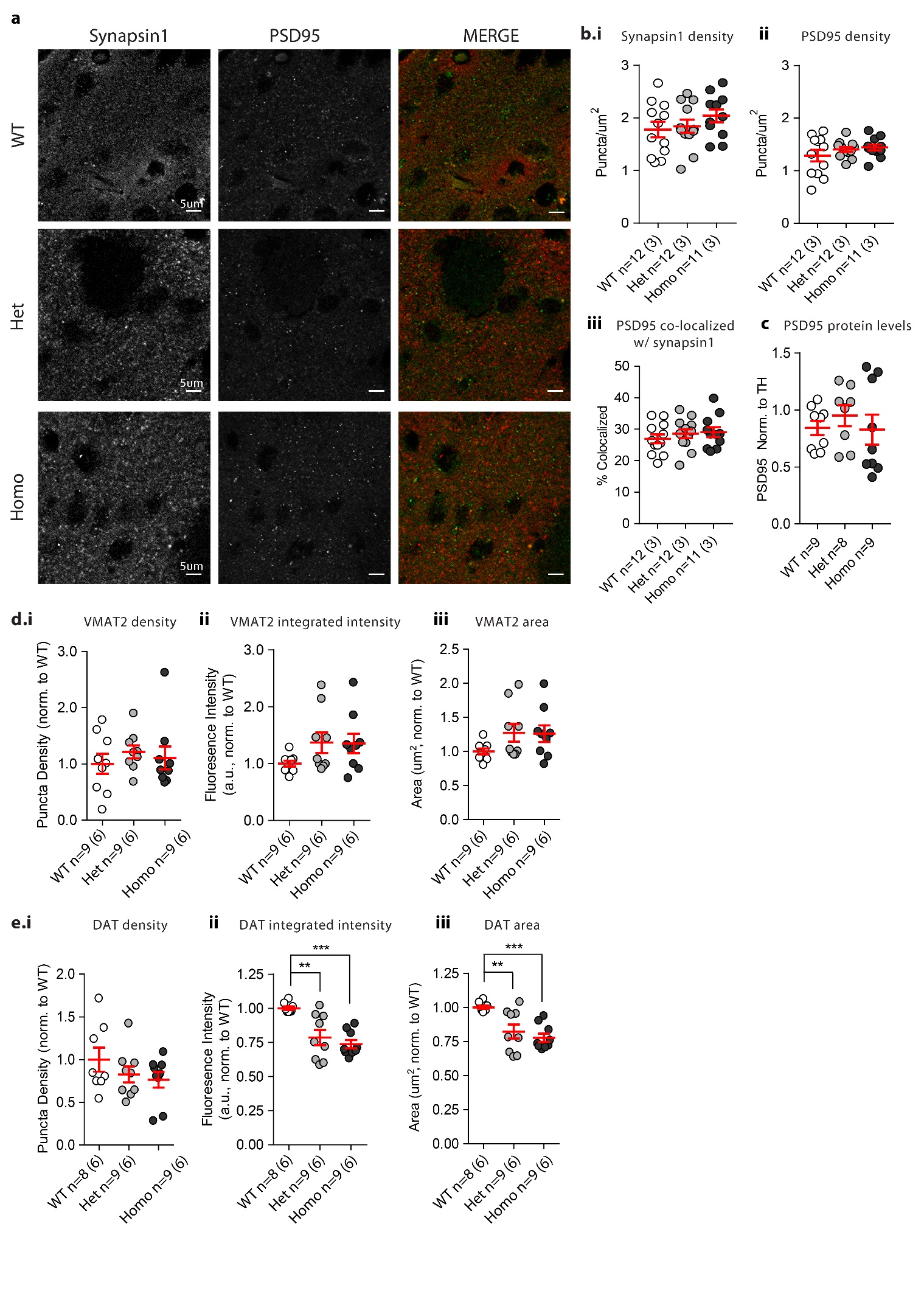
**

**a**) Representative confocal images of synapsin1 (left) and PSD95 (middle), also merged (right), in the dorsolateral striatum of 3-month-old VKI mice. **b**) No changes in either synapsin1 (**b**.i., 1-way ANOVA *F*_2, 32_ = 1.03, *p*=0.36) or PSD95 (B.ii, 1-way ANOVA *F*_2,32_ = 1.14,  *p*=0.33) puncta density were detected, nor in the percentage of synapsin1 and PSD95 co-localization (**b**.iii, 1-way ANOVA *F*_2,32_ = 0.56,  *p*=0.57). **c**) Densitometry analysis was conducted by normalizing the intensity of PSD95 to TH. No difference was found in PSD95 levels (1-way ANOVA *F* _2,23_ = 0.427, *p*=0.65).

**Supp.Fig.5d-e. Synaptic markers synapsin1 & PSD95 in striatal tissue and slices from VKI mice.**


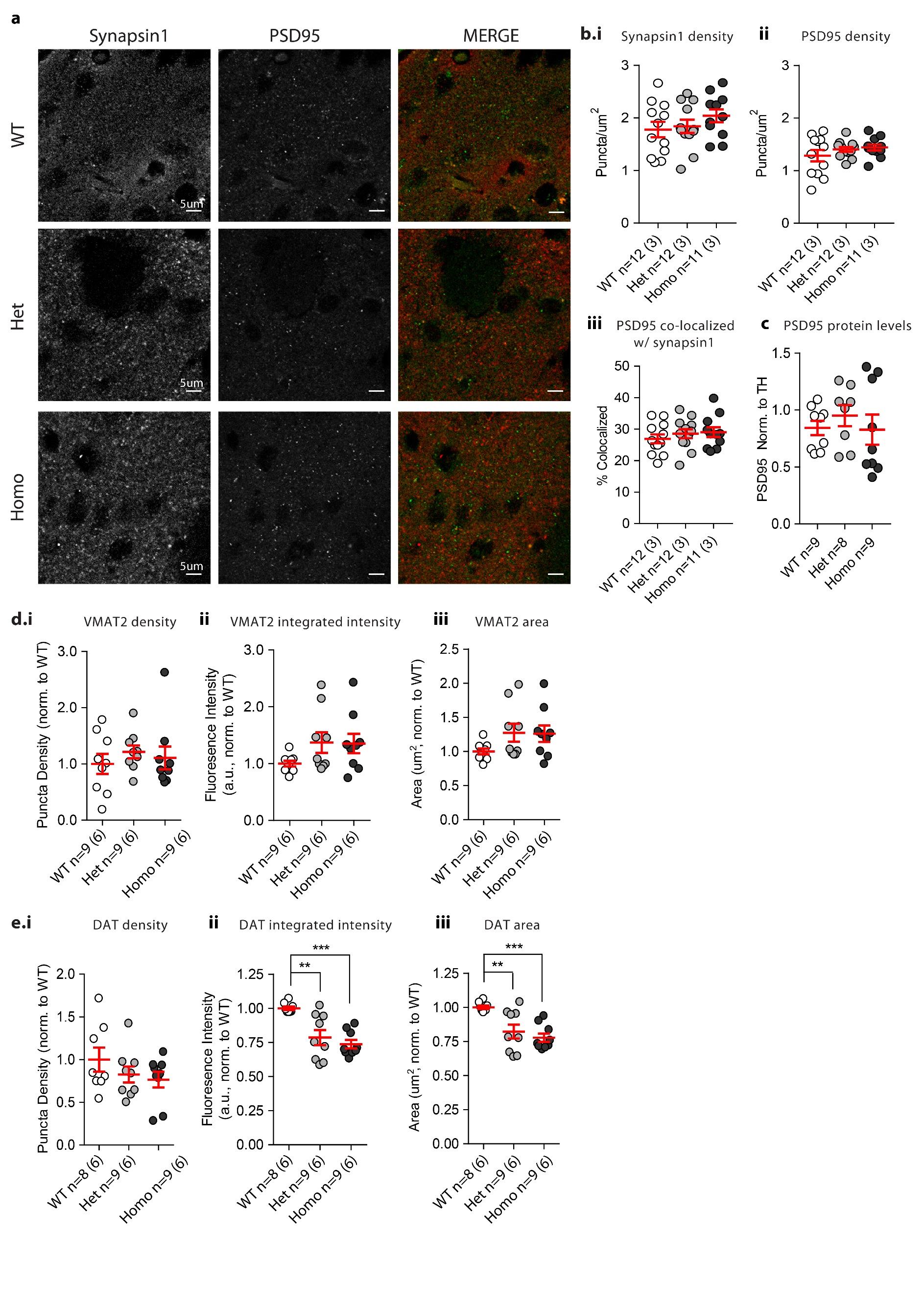


**d-e**) Corresponding graphs of data presented in Figure 6**d** and **e** shown per slice ‘n’ from the total number of animals (indicated in parentheses). **d**) IHC analysis of VMAT2 shows no changes in puncta density (**d**.i, 1-way ANOVA *F* _2, 24_ = 0.392, *p*=0.68), integrated intensity (**d**.ii, 1-way ANOVA, *F* _2, 24_ = 2.01, *p*=0.16) or area (**d**.iii, 1-way ANOVA, *F* _2,24_ = 2.108, *p*=0.14) in VKI compared to WT littermates. **e**) IHC analysis of DAT shows no change in puncta density (**e**.i, 1-way ANOVA *F* _2,23_ =1.21,  *p*=0.31), while the integrated intensity (**e**.ii, 1-way ANOVA, *F* _2,23_ = 12.47 *p*<0.001, *Bonferroni post-test* WT vs Het t_(23)_ = 3.86 *p*<0.01, WT vs Homo t_(23)_ = 4.73 *p*<0.001) and area were significantly decreased in VKI compared to WT littermates (**e**.iii, 1-way ANOVA *F* _2,23_ = 9.94 *p*<0.001, *Bonferroni post-test* WT vs Het t_(23)_ = 3.39 *p*<0.01, WT vs Homo t_(23)_ = 4.25 *p*<0.001). For VMAT2 and DAT immunohistochemistry ‘n’ is the number of image fields.

**Supp.Fig.6 SERT and NET levels in the striatum of VKI mice.**


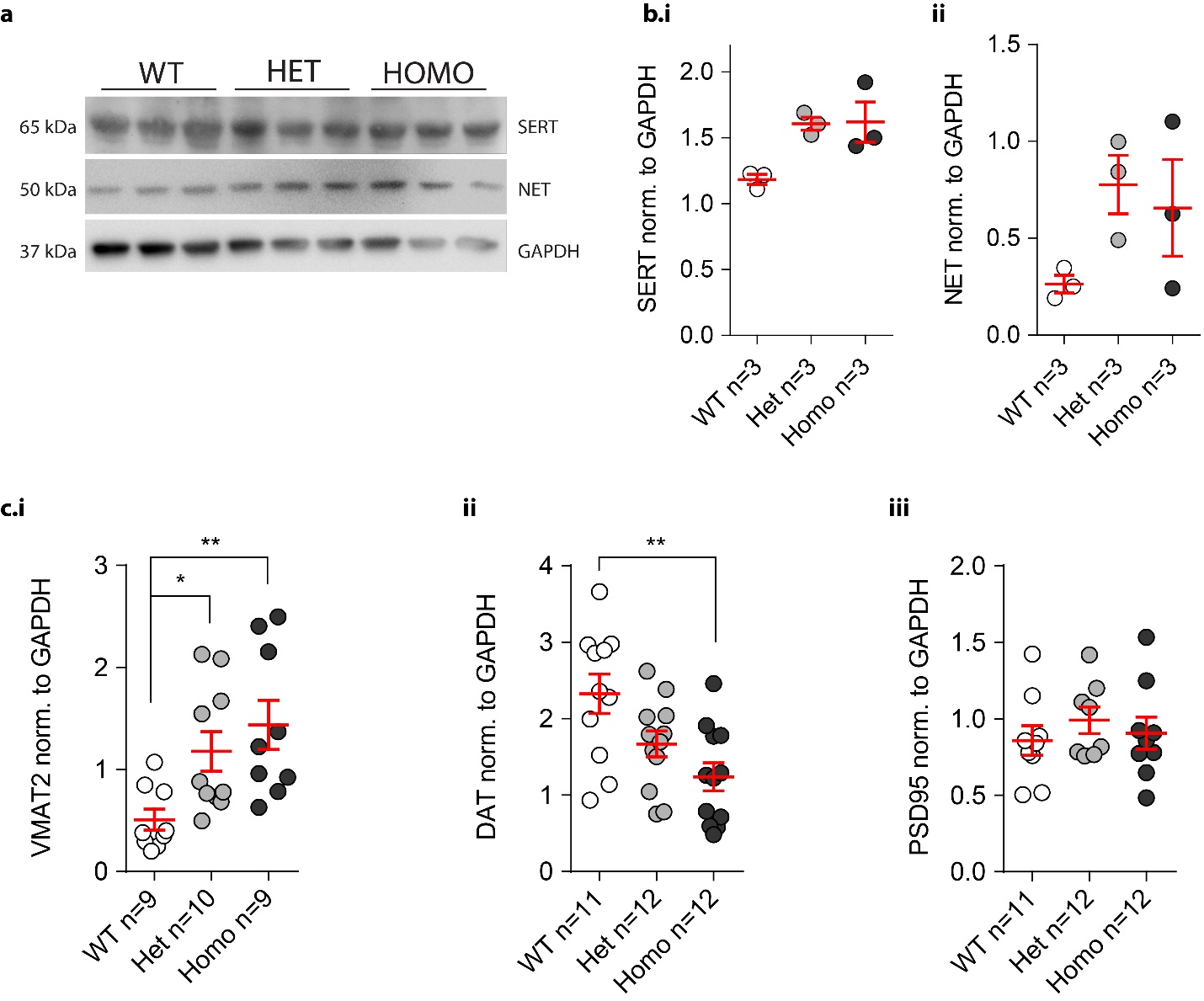


**a**) Representative western blot of SERT and NET in VKI mice. **b**) Densitometry analysis was conducted normalizing SERT and NET intensity to GAPDH loading control. An increase in the mean protein levels of SERT (1-way ANOVA *F*_2,6_ = 6.82, *p* < 0.05, with *Bonferroni post-test* WT *vs* Het t_(6)_ = 3.14, *p* < 0.05 & WT *vs* Homo t_(6)_ = 3.25, *p* < 0.05) was observed in striatal tissue from 3 month old mice, but not NET (1-way ANOVA *F*_2,6_ = 2.49, *p*=0.14). **c**) Protein quantification for VMAT2, DAT and PSD95, shown in Fig.6 and Supp.Fig.5 normalized to loading control GAPDH. VMAT2 is significantly increased in VKI mice (**c**.**i**, 1-way ANOVA *F* _2,25_ = 6.12, p<0.01, *Bonferroni post-test* WT vs Het t_(25)_ =2.51 *p*<0.05, & WT vs Homo t _(25)_ = 3.40, *p*<0.01), while there is a decrease in DAT levels in mutant animals (**c.ii**, 1-way ANOVA *F* _2,32_ = 7.08, p<0.01, *Bonferroni post-test* WT vs Het t_(32)_ = 2.26, *p*=0.06 & WT vs Homo t_(32)_ = 3.74, *p*<0.01). There was no difference in total levels of PSD95 (**c.iii**, 1-way ANOVA *F* _2, 23_ = 0.464, *p*=0.63).

**Supp.Fig.7 D2 levels in striatum**

**
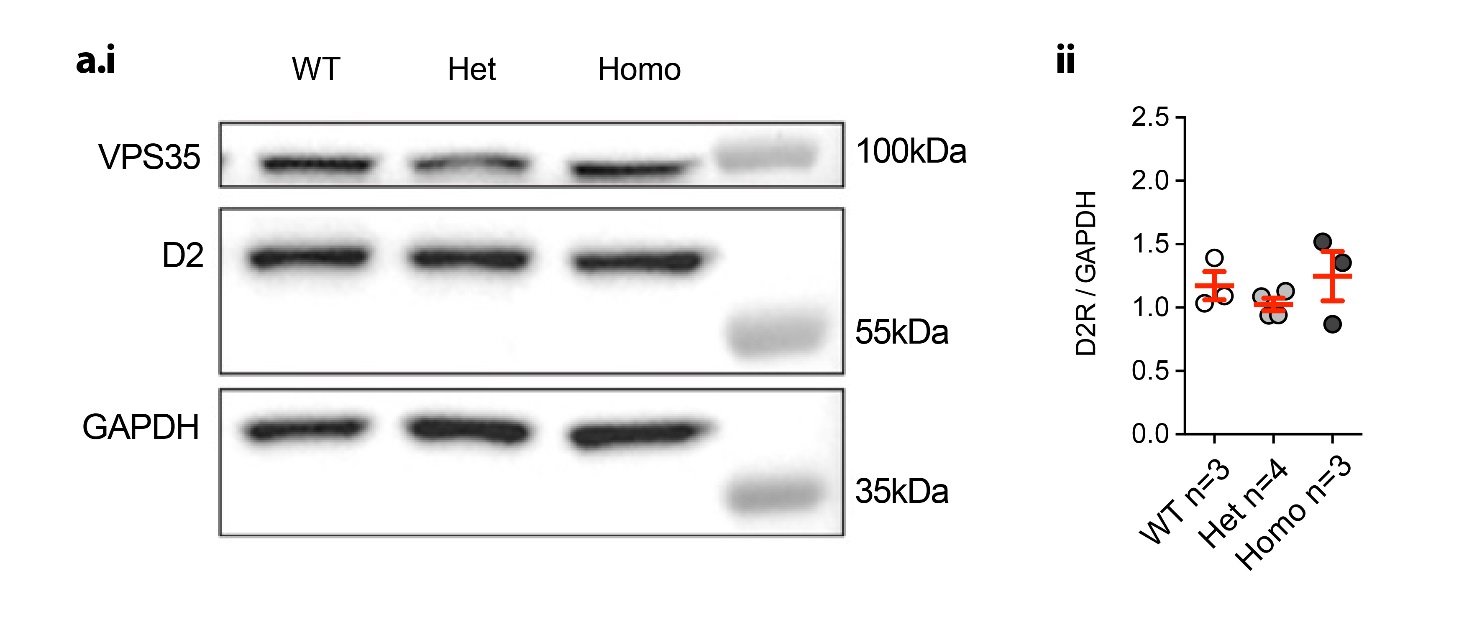
**

**a.i** Western blot analysis was performed on striatal lysates from VKI mice and their littermates using antibodies for D2 receptor, showing no genotype effect on D2 total levels. **a.ii** (1-way ANOVA F_2,15_ = 0.60, *p*=0.56).

**Supp.Fig.8 α-synuclein in striatal tissue and SNpc.**

**
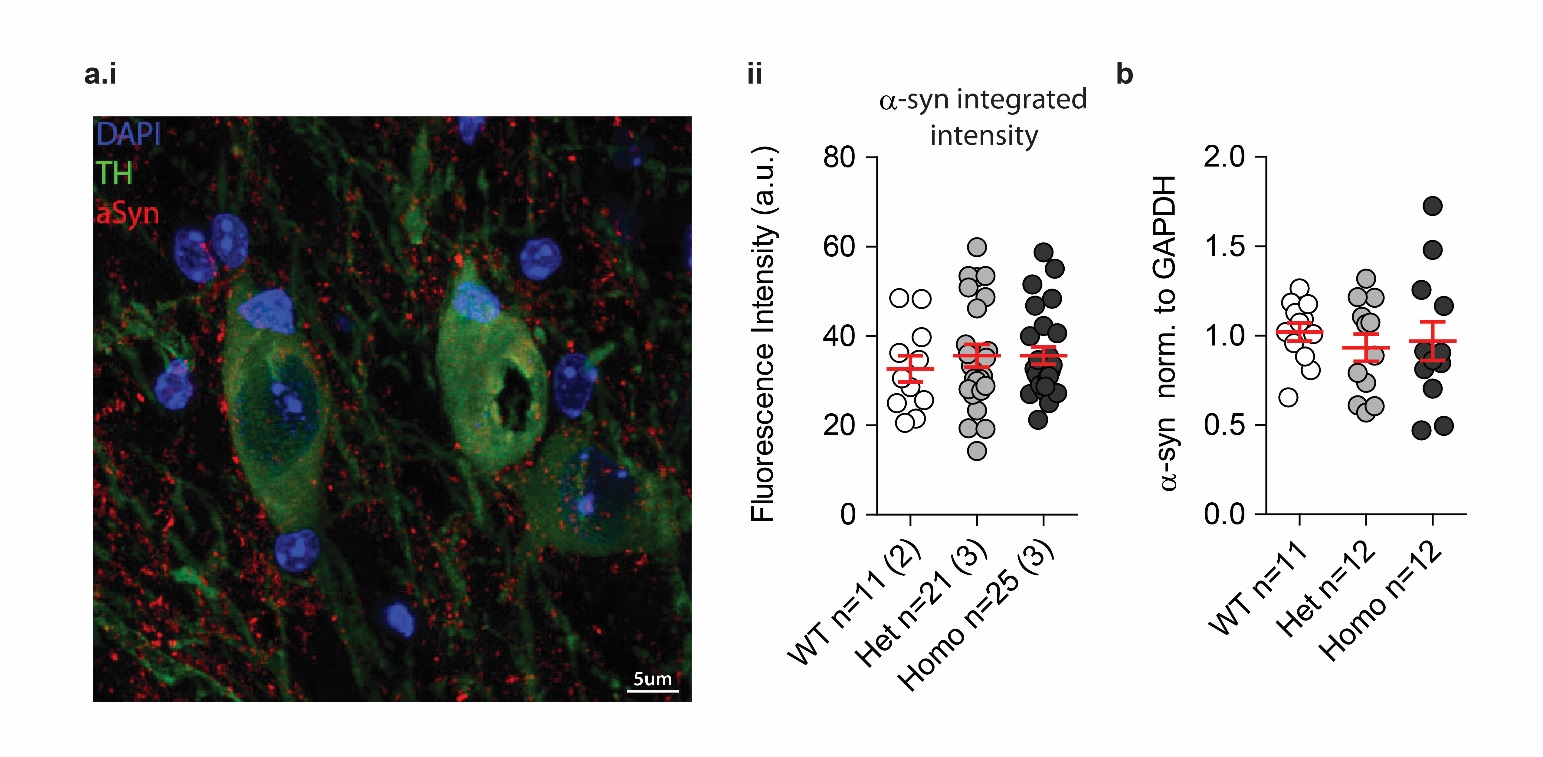
**

**a.i**) Representative image of α-synuclein staining in the SNpc. TH+ neurons are shown in green and α-synuclein in red. a.ii) α-synuclein labelling in the SNpc shows no difference between VKI and WT littermates, quantified from immunohistochemistry (1-way ANOVA F_2,59_ = 0.32, *p*=0.72). **b**) α-synuclein levels in the striatum are comparable in VKI and WT littermates, quantified by western blot (1-way ANOVA *F*_2,33_ = 0.28, *p*=0.75).

**Supp.Table 1. Primers sequences of *VPS35, Actb, Gapdh* and *Rpl19*.**

| **Primer** | **Sequence 5′ to 3′** | **Tm** | **Gene name** |
| --- | --- | --- | --- |
| *Vps35* forward | AAGATGGACCCGGAATTCC | 60 | Vacuolar protein sorting 35 |
| *Vps35* reverse | GTAGTCCACACGATCAGGG |  |  |
| *Actb* forward | GATCTGGCACCACACCTTCT | 60 | Actin beta |
| *Actb* reverse | CCATCACAATGCCTGTGGTA |  |  |
| *Gapdh* forward | CTTTGGCATTGTGGAAGGG | 60 | Glyceraldehyde-3-phosphate dehydrogenase |
| *Gapdh* reverse | TGCAGGGATGATGTTCTGG |  |  |
| *Rpl19* forward | AATCGCCAATGCCAACTC | 60 | Ribosomal protein L19 |
| *Rpl19* reverse | GGAATGGACAGTCACAGG |  |  |
